# Supplementary material for: Verification and execution of the scientific literature via chemputation augmented by large language models
Source: Commun Chem. 2026 Apr 3;9:191. doi: 10.1038/s42004-026-01993-w (PMC13219472; doi:10.1038/s42004-026-01993-w)
Supplement: Supplementary file 2 — SI [file 42004_2026_1993_MOESM2_ESM.pdf]

Supporting Information for:

Verification and Execution of the Scientific Literature via Chemputation Augmented by Large Language Models

Sebastian Pagel, Michael Jirasek, Leroy Cronin*.

*Lee.Cronin@glasgow.ac.uk

School of Chemistry, The University of Glasgow, University Avenue, Glasgow G12 8QQ, UK.

Contents

1 ACRA details ... 4

1.1 Agents ... 4

1.1.1 Paper Scraper Agent ... 5

1.1.2 Procedure Agent ... 8

1.1.3 Ambiguity Agent ... 14

1.1.4 *X*DL-Agent ... 15

1.1.5 Critique Agent ... 20

1.2 Long-term storages ... 23

1.2.1 Priming of the Chemical Ambiguity Database ... 23

1.2.2 Priming of *X*DL Database ... 24

1.2.3 Structure of Paper Knowledge Graph ... 24

1.2.4 *Labbook* structure ... 25

1.2.5 Optional Expert Input ... 26

1.2.6 Filling in Missing Chemical Information ... 26

1.3 Error Capturing ... 27

1.3.1 *X*DL-Validity ... 27

1.3.2 Discrepancy Check (LLM-as-a-judge) ... 28

1.3.3 Simulation of Execution ... 28

1.3.4 Overall Translation Efficiency ... 29

1.4 Parsing of Textual Documents ... 29

2 Benchmarks ... 30

2.1 Benchmarking the classification of extracted literature procedures by the Procedure Agent ... 30

2.1.1 Benchmark Data ... 30

2.1.2 Benchmark Results ... 32

2.1.3 Sanitization of Literature Procedures ... 34

2.2 Knowledge Graph Extraction ... 35

2.2.1 Methodology ... 35

2.3 *X*DL Translation ... 37

2.3.1 Methodology ... 37

2.4 Procedures for Benchmarking Translation Efficiency ... 37

2.5 Procedures for the Benchmark of Memory Components ... 39

2.6 Output comparison of different models ... 41

3. Comparison to previous projects ... 44

4 Reaxys, ORD, and analysis, not executable steps 45
4.1 Procedure Keyword Analysis from the Reaxys Database 45
4.2 Most frequent n-grams in the Open Reaction Database 46
4.3 Analysis of not executable steps 46
5. Details of performed synthesis 48
5.1 Hardware 48
2.2.1 Methodology 48
2.2.1 Methodology .... **Error! Bookmark not defined.**
5.1 Synthesis of 3-Methoxy-3-oxopropanoic acid 51
5.2 Synthesis of p-toluenesulfonate 54
5.3 Synthesis of 2-Methyl-2-(3-oxopentyl)-1,3-cyclohexanedione 57
5.4 Synthesis of Methyl 4,6-O-benzylidene-α-D-glucopyranoside 61
5.5 Synthesis of (2E)-3-[3,4-bis(acetyloxy)phenyl]-2-propenoic acid 64
5.6 Synthesis of 4-(4-nitrophenyl)morpholine 67
References 70

1 ACRA details

The proposed system, Autonomous Chemputer Reaction Agents (ACRA), was designed as a multi-agent workflow in which specialized agents interact to validate/reproduce chemical synthesis literature, all the way from parsing a (primary) literature source, to execution of the synthesis on a robotic platform. Starting from a literature source (i.e. scientific publication) the workflow is as follows:

Parse text → Extract and Combine Data → Include Chemical information (unify names, include properties, boiling point, etc.) → resolve ambiguities → translate literature procedure to *X*DL → validate *X*DL → execute synthesis → compare analytical data to extracted analytical data

In the following sections, each of the components in the workflow is described in detail.

Model versions:

- *gpt-4o:* gpt-4o-2024-05-13
- *gpt-4o-mini:* gpt-4o-mini-2024-07-18
- Embeddings: text-embedding-3-large

1.1 Agents

ACRA is composed of multiple specialized agents that work together to extract synthesis information from literature, resolve ambiguities in extracted procedures, and translate and validate using the Chemical Descriptor Language (*X*DL). Synthesis (and related) information is retrieved from a literature source by the scraping agent. It iteratively extracts data from chunks of a literature source and creates a unified knowledge graph (KG). Extracted procedures are then sanitized and checked for ambiguities by the Procedure-Agent. Previously resolved ambiguities are provided for guidance. Finally, sanitized procedures are translated into *X*DL by

the *X*DL-Agent and validated in a three-step workflow. First, the *X*DL is validated for syntactic validity. Subsequently, the Critique-Agent checks for discrepancies between the generated *X*DL and the initial procedure. Finally, *X*DLs that pass the first two stages are mapped to a predefined hardware graph, and the execution is simulated. All errors encountered during any of these stages are fed back to the *X*DL-Agent with instructions to resolve those (Figure S1).

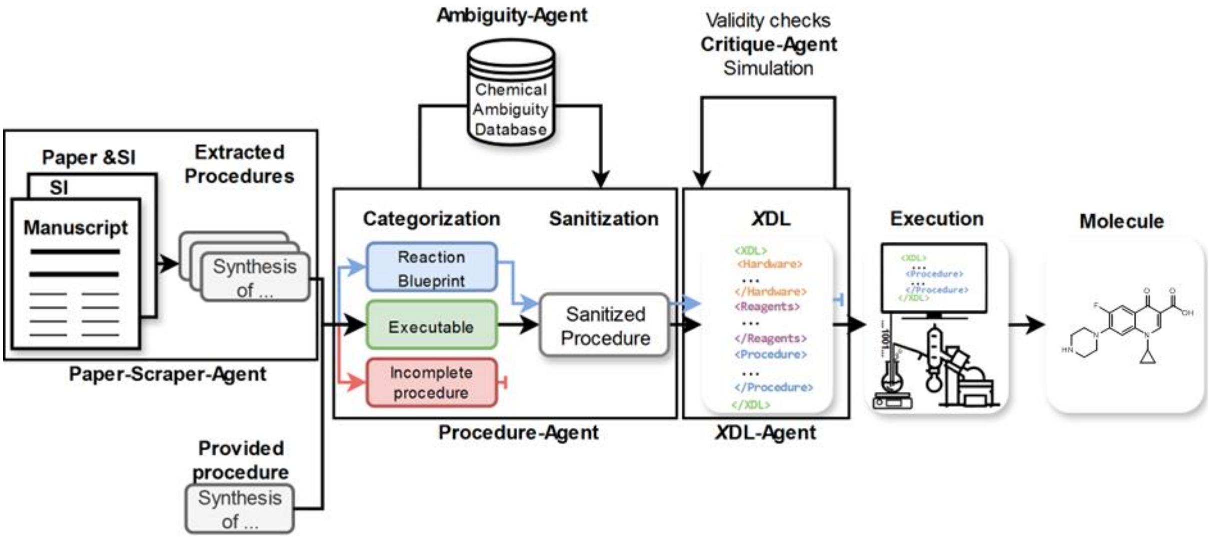

Figure S1: Abstract depiction of the workflow implemented in ACRA.

1.1.1 Paper Scraper Agent

The Paper-Scraper-Agent (scraping-agent) is implemented using the gpt-4o-mini model from OpenAI and takes a set of documents (e.g. main manuscript and supporting information) and is instructed to extract all synthesis-related information from the text in a predefined format (compare Figure S4). First, all textual data is combined into a single text document and subsequently split into chunks of 4096 tokens using the cl100k_base tokenizer. Each chunk is set to have 1024 overlapping tokens with the previous and subsequent chunks to ensure the entire context of each text part will be present in at least one chunk. Every chunk is then iteratively provided to the Paper-Scraper-Agent with an initial prompt to extract synthesis-

related data (procedures, chemical names, purification, analytical data, and additional information) from a given text chunk (compare Figure S2 and S4). The agent is instructed to return its response in a JSON format. Subsequent text chunks are treated similarly (Figure S3). In addition to the current chunk of the text, the titles of the already extracted procedures were provided to guide the extraction from subsequent text chunks.

To reduce cost and latency, we settled on using gpt-4o-mini for the extraction of data from literature sources. Additionally, while the context window (number of tokens per prompt) for both gpt-4(o) and gpt-4o-mini allows a substantially larger number of tokens than the 4096 provided during this study, we experienced that extraction strongly deteriorated when extracting large amounts of structured data from longer texts, often because of the limited number of output tokens (4096 for gpt-4(o)). The extended number of output tokens of gpt-4o-mini (16384 tokens) was thus favorable in this case.

In addition to extracting information from the provided document(s), the textual data was embedded into a vector database in chunks of 2048 tokens using the OpenAI model *text-embedding-large*. This vector database was later used during the sanitization of extracted procedures by the Procedure-Agent.

````
procedure_scraper_prompt = """
This is a scientific paper that might contain procedures for synthesizing new compounds:
```
{paper}
```

To respond do the following:
    {INSTRUCTIONS}

There might be no procedures in the paper.
Before you respond make sure to carefully read the paper, extract all relevant
information, and carefully read the instructions.
Respond with the full procedure with all abbreviations filled in.
Use the following json format:
```
{response_format}
```

Only extract synthetic procedures for which a detaild procedure is given (i.e. the synthetic steps are given).
Never repeat the same information twice.
Do not abbreviate anything, I dont have hands and cannot look up the paper.
"""
````

Figure S2: Prompt template used for the extraction of synthesis-related data from the initial text chunk provided to the Paper-Scraper-Agent.

```
iterative_procedure_scraper_prompt = """
Previously you have extracted the following information for the following procedures:
```
{previous_response}
```

The next section of the paper is the following:
```
{paper}
```

Try to extract any information from the paper associated with the procedures:
    {INSTRUCTIONS}

There might be no procedures in the paper.
Before you respond make sure to carefully read the paper, extract all relevant
information, and carefully read the instructions.
Respond in the json following format:
```
{response_format}
```

Only extract synthetic procedures for which a detaild procedure is given (i.e. the synthetic steps are given).
Never repeat the same information twice.
Do not abbreviate anything, I dont have hands and cannot look up the paper.
"""
```

Figure S3: Prompt template used for the extraction of synthesis-related data from every chunk after the first one.

```
response_format = """
{
    ""
    "chemicals": {
        chemical_name: [abbreviation/synonym, abbreviation2/synonym2, ...,],
        chemical_name: [abbreviation/synonym, abbreviation2/synonym2, ...,],
        ...
    },
    "procedure_titles": [list of all chemical procedure titles in the paper],
    "procedure_texts": {
        "procedure_title": {
            "full_procedure_text": ...,
            "product": ..., "chemicals": [...],
            "purification": {purification_method: [purification_data], purification_method: [purification_data], ...},
            "yield": percentage_yield,
            "analysis": {analysis_method_name: [analysis_data], analysis_method_name: [analysis_data], ...},
            "additional_info": [all relevant additional info]},
        ...
    },
}
"""
```

Figure S4: Response instructions for the Paper-Scraper-Agent for every text chunk.

1.1.2 Procedure Agent

Extracted procedures were sanitized by the Procedure-Agent to resolve chemical ambiguities. This includes resolving chemical names, amounts defined in incompatible units, vaguely defined times, etc. First, the Procedure-Agent was instructed to identify all chemicals and their amounts in the given procedure. Additionally, potential hazards should be identified. To resolve ambiguities in the procedure, a set of previously resolved ambiguities were included in the prompt that were resolved for a similar procedure, extracted via semantic similarity search (see Ambiguity-Agent below). A set of common ambiguities (Figure S9) was provided for each translation regardless of the procedure. The Procedure-Agent was then instructed to identify all ambiguities in the procedure that it can resolve itself, given the provided information and its general reasoning capabilities, as well as ambiguities it cannot resolve itself and note both down (Figures S5 and S6).

The identified chemical names were attempted to be unified by searching for information using the PubChem database. Since the PubChem database summarizes not just IUPAC names, but also commonly used abbreviations and other non-IUPAC names, this allows the identification

of some molecules with non-standardized names (1,4-Diazabicyclo[2.2.2]octane is captured with 125 synonyms, for example). Physical units of extracted chemicals were attempted to be unified (e.g. milligrams → grams, mol → grams etc.) for molecules that were identified via the PubChem search. A solvent database[1] was used to provide physicochemical data (boiling point, melting point and density) for extracted solvents within the context of the Procedure- and XDL-Agents. The identification of molecules via the PubChem search, the conversion of physical units, and the extraction of physicochemical data were not controlled by an LLM (e.g. via function calling[2,3]), but instead programmatically given the extracted chemical names and amounts in a structured format (compare Figure S6).

```
procedure_prompt = """
This is the procedure paper you are working on:
``` {procedure} ```

Commonly found ambiguities in chemical synthesis procedures are:
```{ambiguities} ```

Additionally here are some examples of ambiguous language and their solutions
from previous procedures that you might find helpful:
``` {previous_ambiguities} ```

Please do the following:
    1. Identify all chemicals used in the procedure.
        - Include the chemical name, amount, and unit.
        - If the name is not in english, please translate it to english.
        - Only include the chemical names, and strip any extra information (e.g.
          "anhydrous" or "dry" or "soluion", or "concentration", etc.)
        - do not extract chemical if their name is unspecific like "reagent A",
          "Aldehyde 1", "Ketone 5", "reagent B", "compound A", "A", "5d", "1a"
          etc.
    2. Identify all potential hazards in the procedure.
        - This includes physical and chemical harms, environmental hazards, and
          safety concerns.
        For example: - High pressure - Air sensitive reagents - Toxic reagents -
        Etc.
    3. Identify all ambiguities you think you can resolve yourself. Use the
       ambiguity library and provided examples to resolve ambiguities as well as
       your reasoning capabilities (guesses for physical values should always
       depend on the procedure and the amount, conditions, etc used).
        - ambigious or unclear language, and ambiguous or unclear steps.
        - missing information
        - unclear conditions (e.g. temperature, pressure, time, etc.)
        - unclear amounts (e.g. stoichiometry, equivalents, drops, etc.)
        - ambigious steering speeds
    4. Write down all ambiguities that you CAN NOT resolve yourself/lines where
       the order of execution is unclear. Even after consulting the ambiguity
       library and provided examples, you are still unsure about the meaning of
       the line.
        - this concerns lines where you are not sure about the order of execution
            or the meaning of the line.
        - this also concerns lines where you are not sure if an error is present
            in the representation of the procedure.

Respond in the following valid json format: ``` {response_format} ```
"""
```

Figure S5: Prompt template used for the extraction of chemicals, hazards, as well as ambiguities for a given procedure.
```

```
response_format = """
{
    "ambiguities": {"ambiguity1": "solution", "ambiguity2": "solution", ...},
    "unresolved_ambiguities": {"full sentence from procedure here": {
    "questions": [
        "question 1",
        "question 2",
        ...
    ]
    }, "full sentence from procedure here": {
    "questions": [
        "question 1",
        "question 2",
        ...
    ]
    }}, # if no unresolved ambiguities, write an empty dictionary
    "chemicals": [[name, amount_used_in_procedure, unit], [name,
    amount_used_in_procedure, unit], ...], # If no amount is provided in the
    procedure, write [name, None, None]
    "hazards": [List of potential hazards identified in the procedure. If None,
    write an empty list],
}
"""
```

Figure S6: Response format for the prompt in **Figure S5**.

Ambiguities that were identified by the Procedure-Agent and could not be resolved were optionally resolved via expert input (see below).

To generate sanitized procedures, the initial procedure, the identified chemicals with their converted amount and extracted additional data were provided to the procedure agent. Alongside that, all identified resolved ambiguities, previously resolved ambiguities from the ambiguity agent, and commonly identified ambiguities were provided. The Procedure-Agent was then instructed to generate a new, sanitized procedure description, list all unresolved ambiguities, and categorize the procedure into A (*executable)*, B (reaction *blueprint)*, or C (*not executable/*having *missing information;* compare Figures S7 and S8). If any chemical amounts were not resolvable, the classification was automatically set to B, unless the Procedure-Agent classified it as C. The Procedure-Agent was implemented with the OpenAI model *gpt-4o* and

a *temperature* setting of 0. In case an error occurred during either step, the respective step was repeated with a temperature setting of 0.05.

````
clean_procedure_prompt = """
The following information was provided by the user. Always obey the user's information.:
```
{user_request}
```

The procedure was as follows:
```
{procedure}
```

The chemicals used in the procedure are:
```
{identified_chemicals}
```

The ambiguities identified in the procedure are:
```
{identified_ambiguities}
```

Additionally the following ambiguities were resolved by an expert:
```
{ambiguity_library}
```

To resolve the ambiguities feel free to use this knowledge if helpful.
Ambiguities that are typically found in chemical synthesis procedures are:
```
{ambiguities}
```

Additionally here are some examples of ambiguous language and their solutions
from previous procedures:
```
{previous_ambiguities}
```

Please do the following:
    {RESPONSE_INSTRUCTIONS}

Before you start note down all changes you make to the procedure and the reasons for the changes.
For example:
1. Changed: "added rapidly" to "added"
   Reason: "added rapidly" is ambiguous and can be interpreted in different ways.
2. Changed: "ambient temperature" to "25°C"
   Reason: "ambient temperature" is ambiguous and can be interpreted in different ways.
3. Changed: 2 mmol to 1.06 g
   Reason: 2 mmol can not be easily measured, so it is better to use the mass of the chemical.


Respond to the following valid json format:
```
{response_format}
```
"""
````

Figure S7: Prompt template for the generation of sanitized procedures.

```
response_format_clean_procedure = """
{
    "procedure": "Rewritten procedure",
    "chemicals": [list of chemicals used in the procedure],
    "remaining_ambiguities": [list of remaining ambiguities identified in the
    procedure. If none, write an empty list],
    "classification": {
        "reasoning": "Reasoning for the classification according to the
        definitions provided above",
        "classification": "A or B or C",
    }
}
"""
```

Figure S8: Response format for the generation of sanitized procedures.

```
common_ambiguities = """
solution of strong acid in water: first add water to vessel, then add acid to vessel
reflux/ was refluxed/ ...: mixture should be heated to boiling point of the solvent while stirring,
A was dissolved in solvent B/a solution of x in y was prepared: Add A to vessel,
then add B to vessel while stirring. Stir for some time afterwards before
proceeding,
chemical was dissolved in solvent/ was dissolved/ ...: mixture should always be
stirred for some time (so stir= True) after adding solvent,
pressure identical: in vacuo == under vacuum == under reduced pressure ==
solvent was evaporated == evcuated,
stirred vigorously: 1000 rpm,
stirred rapidly: 1000 rpm,
stirred: 300 rpm,
added rapidly == added,
added over X minutes == added dropwise,
temperature range (i.e. 80-90°C): one exact temperature should be given,
ambient temperature: 25°C,
room temperature: 25°C,
1 drop is equivalent to 0.05 mL,
overnight: 12 hours,
all times, temperature, pressure, and speed conditions should exactly quantified,
solution of A (solid) in B (solvent)/ dissolved in solvent: add solvent to
vessel and stir for some time to allow for dissolution,
recrystallization/recrystallize: add solvent to vessel containing the solid and
heat to boiling point of solvent, after strring for some time to allow for
dissolution, cool to room temperature (unless otherwise stated). Subsequently
evaporate to remove solvent (This means it should always be executed in a vessel
that can be evaporated/dryed),
extracting or washing: There always needs to be a phase separation. This means
that usually a aqueous and organic solvent are used.
"""
```

Figure S9: Common chemical ambiguities provided within every prompt of the Procedure-Agent

1.1.3 Ambiguity Agent

The Ambiguity-Agent was used to initialize the Ambiguity-Database described below. 5 synthetic procedures were given to the Ambiguity-Agent and instructed to ask three questions about every sentence in the procedure (Figure S10). All questions were carefully answered by an expert chemist, and the resolved ambiguities alongside the answers were stored in a vector database (Chemical Ambiguity Database; CAD). The questions asked by the Ambiguity-Agent were used as keys in the CAD by embedding the text using the OpenAI model text-embedding-large. The ambiguities that the Procedure-Agent identified and could not resolve were stored in the CAD if additional expert input was used to resolve them. This way, more and more ambiguities could be resolved and thus available for subsequent procedures.

```
ambiguity_prompt = """
This is a chemical synthesis procedure that you need to make unambiguous and reproducible:
{procedure}

Now ask questions about each step of the procedure to resolve any ambiguities.
These can include:
- everything that needs some unit to be performed (e.g. mL, g, etc.)
- unclear conditions (e.g. temperature, pressure, time, etc.)
- unclear amounts (e.g. stoichiometry, equivalents, drops, etc.)
- ambiguous or unlcear order of steps
- and everything else that is unclear or ambiguous

For each step in the procedure, ask at least three question to resolve any ambiguities.
Reply in the following json format:
```
{response_format}
```
"""

response_format = """
{
    "line from procedure here": {
    "questions": [
        "question 1",
        "question 2",
        ...
    ]
    },
    "line from procedure here": {
    "questions": [
        "question 1",
        "question 2",
        ...
    ]
    },
    ...
}
"""
```

Figure S10: Prompt and response format initializing the Chemical Ambiguity Database.

1.1.4 *X*DL-Agent

Sanitized procedures were translated by the *X*DL-Agent into the corresponding *X*DL procedures. Similar to before, an iterative process was used to generate error-free *X*DL procedures[4]. Since error-free *X*DL procedures do not guarantee precise and executable procedures, we greatly expanded on what was presented before. Additionally, we found that previously reported would oftentimes generate *X*DLs that were either not up-to-date with the

current version of *X*DL or were missing sanity checks required to meet the official *X*DL implementation[5].

To generate *X*DLs, the *X*DL-Agent was provided with the documentation of the *X*DL language, similar to what is described in *Skreta et. al.*[4]. The 5 most similar previously translated procedure-*X*DL pairs, the ambiguities described in Figure S9, and previously gathered information about the chemicals used in the procedure. The similarity of previously translated procedure-*X*DLs was calculated via the cosine similarity of the embeddings obtained from the OpenAI model *text-embedding-large* of the procedure texts. To translate, the *X*DL-Agent was instructed to first identify all roles of chemicals in the procedure (e.g. solvent, reagent, catalyst, etc.), then in *ReAct* style-prompting[6], split the procedure into step-by-step instructions, translate the steps into *X*DL, and finally combine all steps into a single *X*DL protocol (compare Figures S11 and S12). We call this prompting scheme *chain-of-abstraction*.

```
xdl_prompt = """
XDL Language Description: ``` {steps_description} ```

Here are examples of chemical procedures in synthetic procedure, and the XDL
code that was used to execute the procedures on a robotic platform: ```
{examples} ```

Additional information In chemistry ambigious language is often used, this can
lead to errors and you should be aware of them: ``` {ambiguities} ```

The synthetic procedure from literature to be translated into XDL is: ```
{new_procedure} ```

Additionally, here is some information of the physical properties of the
chemicals used in the procedure: ``` {chemicals} if a solution contains a salt,
it is assumed to be dissolved in water unless otherwise stated and the density
is 1 g/mL. ```


To respond do the following: ```
    {STEP_BY_STEP_INSTRUCTIONS}
```

Make sure to:
    {INSTRUCTIONS}

Now translate the provided synthetic procedure into XDL. Write down all the
steps. I dont have hands and cant read your mind.
"""
```

**Figure S11:** Prompt template of the *X*DL-Agent for initial generation of a *X*DL from a given procedure.
```

```
chemicals = """
{
   chemical_name_1: {
      "role": "role of the chemical (i.e. solvent, reagent, catalyst, substrate,
      acid, base, activating-agent, product, ...)", "amount": "amount of the
      chemical", },
   chemical_name_2: {
      "role": "role of the chemical (i.e. solvent, reagent, catalyst, substrate,
      acid, base, activating-agent, product,s ...)", "amount": "amount of the
      chemical",},
   ...
}
"""


step_by_step_instructions = """
{
   "step_1": {
      "thought": what you think the first step in the synthetic procedure is,
      "reasoning": why you think this is the first step, "instruction": first
      step in the synthetic procedure, "xdl_step": XDL step for the first step
   },
   "step_2": {
      "thought": what you think the second step in the synthetic procedure is,
      "reasoning": "why you think this is the second step", "instruction":
      "second step in the synthetic procedure", "xdl_step": "XDL step for the
      second step"
   },
   ...
}
```

Figure S12: Response instructions for the initial generation of *X*DL

The generated *X*DL was validated with the above-described workflow. All errors identified were collected and provided to the *X*DL-Agent alongside the previous *X*DL and all previously mentioned information (Figure S13). To correct the *X*DLs, the *X*DL-Agent was instructed to first map the identified errors to the corresponding lines in the *X*DL and provide a correction. Additionally, the *X*DL agent was instructed to identify disordered steps that do not match the procedure and correct them (Figure S14). Finally, the *X*DL-Agent was instructed to return an updated *X*DL with all the corrections. This process was repeated until the generated *X*DL passed

all three stages of the validation pipeline, or reached a maximum number of iterations (6 if not mentioned otherwise).

The *X*DL-agent was implemented with the OpenAI model *gpt-4o* and a temperature setting of 0 for both the initial and iterative generation.

```
iterative_xdl_prompt = """
XDL Language Description: ``` {steps_description} ```

This synthetic procedure: ``` {new_procedure}

and chemical information: {chemicals} if a solution contains a salt, it is
assumed to be dissolved in water unless otherwise stated and the density is 1
g/mL. ```


Was translated into this uncorrect XDL code: ``` {old_xdl} ```

These were the errors: ``` {errors} ```

Additional information: In chemistry ambigious language is often used, this can
lead to errors: ``` {ambiguities} ```

Please fix the errors and warnings. While you are correcting the new XDL code,
make sure to check for any potential errors that might not have been captured in
the previous execution.

To respond do the following: ```
   FIRST:
      Map the errors and warnings to all lines of the XDL code of the synthetic
      procedure causing the error and correct them.
         {error_mapping_format}

   FINALLY:
      Correct the provided XDL with the inconsistencies and error identified
      above. Do not change anything else. Stricltly follow the format of the XDL
      language: ```XDL <XDL> ... </XDL> ```
```

Make sure to:
    {INSTRUCTIONS}

I dont have hands and cant read your mind.
"""
```

**Figure S13:** Prompt template for the iterative generation of *X*DL.
```

```
misordered_steps = """
{
   "inconsistent_steps_1": {
      "line_in_procedure": "line of the synthetic procedure that is not properly
      represented in the XDL code", "xdl_line": "line of the XDL code where the
      step should be", "correction": "correction to the XDL code"
   }, "inconsistent_steps_2": {
      ...
   }
}
"""

error_mapping_format = """
{
   "error_1": {
      "error": "error description", "xdl_line": [lines in the XDL code where the
      error is present], "correction": [correction of each line that contains
      the error]
   }, "error_2": {
      ...
   }
}
"""
```

Figure S14: Response format for the iterative generation of *X*DL.

1.1.5 Critique Agent

During the *X*DL validation process, the Critique-Agent was implemented to identify discrepancies between the procedure and the *X*DL it was translated to. This proved to be an important part of the validation process since syntactic validation alone does not ensure accuracy (compare main manuscript Figure 4D). The Critique-Agent was provided with a description of the *X*DL language, the initial procedure, and the chemicals and extracted additional information from the Procedure-Agent as well as the generated *X*DL (compare Figure S15). The Agent was then instructed to identify any missing or incorrectly implemented steps that did not align with the procedure. Additionally, the Agent was instructed to identify steps that are currently not supported within *X*DL and can thus not be appropriately translated (Figure S16). In both cases, the Critique-Agent was instructed to follow a *ReAct* response

format. Subsequently, the Critique-Agent was instructed to implement the identified missing/ not properly translated steps (Figure S17). The Critique-Agent was implemented with the OpenAI model *gpt-4o* and a temperature setting of 0.

```
critique_prompt = """
Implemented steps in the XDL language (If something is not defined here, it is
not available in the XDL language):
```implemented steps in the XDL language
{steps_description}
```

natural language description of the synthetic procedure. You should evaluate the
correctness of the XDL of the synthetic procedure based on this description:
```natural language description of the synthetic procedure
{procedure_description}

and chemical information:
{chemicals}
if a solution contains a salt, it is assumed to be dissolved in water unless
otherwise stated and the density is 1 g/mL.
```

This is the XDL of the synthetic procedure the natural language description of
the synthetic procedure was translated into:
```XDL Code
{code}
```

Please evaluate the correctness of the XDL of the synthetic procedure. Identify any inconsistencies
between the natural language description and the XDL of the synthetic procedure. Highlighting any
missing steps or errors in the XDL of the synthetic procedure, and group them by `not_executeable`
[step not executeable in XDL language] and `missing_steps` [missing in procedure
but executeable]. Provide suggestions on how to improve the XDL code.
Make sure to:
    {RESPONSE_INSTRUCTIONS}

The response format should strictly follow this
json format:
{response_format}

If everything is correct, just respond with an empty json object
Make sure to strictly stick to the descirbed output json format
"""
```

Figure S15: Prompt template of the Critique-Agent to identify missing and non-executable steps.

```
response_format = """
{
    "not_executable": [
        {
            "thought": which part of the natural language procedure you think is
            not executable with the XDL language, "interpretation": what you
            think this part means to achieve, "reasoning": why you think it is
            not executable with the current XDL language, "suggestion":
            suggestion to improve the XDL language and what to do (if
            applicable),
        }, {
            "thought": which part of the natural language procedure you think is
            not executable with the XDL language, "interpretation": what you
            think this part means to achieve, "reasoning": why you think it is
            not executable with the current XDL language, "suggestion":
            suggestion to improve the XDL language and what to do (if
            applicable),
        },
        ...
    ], "missing_steps": [
        {
            "thought":  which part of the natural language procedure you think
            is missing or incorrectly translated to XDL of the synthetic
            procedure, "natural_language_procedure_line": the actual text in the
            natural language procedure where you think the step is missing or
            incorrect, "reasoning": why you think this step is missing or
            incorrect in the current XDL of the synthetic procedure,
            "suggestion": correction that should be made in plain language,
            "xdl_step": corrected, added, or reordered lines that should be
            included/changed in the XDL of the synthetic procedure (ONLY steps
            and attributes from the XDL language!),
        }, {
            "thought":  which part of the natural language procedure you think
            is missing or incorrectly translated to XDL of the synthetic
            procedure, "natural_language_procedure_line": the actual text in the
            natural language procedure where you think the step is missing or
            incorrect, "reasoning": why you think this step is missing or
            incorrect in the current XDL of the synthetic procedure,
            "suggestion": correction that should be made in plain language,
            "xdl_step": corrected, added, or reordered lines that should be
            included/changed in the XDL of the synthetic procedure (ONLY steps
            and attributes from the XDL language!),
        },
        ...
    ],
}
"""
```

Figure S16: Response format from the Critique-Agent to identify missing and not executable steps.

```
inclusion_prompt = """
natural language description of the synthetic procedure. This is the ground
truth that should be translated into XDL code:
```natural language description of the synthetic procedure
{procedure_description}
```

This is the XDL of the synthetic procedure the natural language description was translated into:
```xdl_code
{code}
```

You were requested to incorporate the correction into the XDL of the synthetic
procedure the natural language description:
```
{requested_correction}
```

Respond with the corrected XDL of the synthetic procedure that incorporates the
requested correction.
Only make adjust the parts of the XDL necessary to incooperate the requested
changes.
Write the enitre XDL of the synthetic procedure. Do not abbreviate anything!
"""
```

Figure S17: Prompt template for inclusion of the identified missing steps by the Critique-Agent.

1.2 Long-term storages

1.2.1 Priming of the Chemical Ambiguity Database

The Chemical Ambiguity Database (CAD) was initialised by requesting the Ambiguity-Agent to ask 3 questions for every step in the literature procedure. Each question was answered in detail by a trained chemist, and each question was used as an embedding in a vector database. Embeddings were generated with the model *text-embedding-large* from OpenAI. At the stage of sanitizing the literature procedure, each sentence of the procedure was embedded, and the two most similar, previously explained sentences were provided within the prompt. The similarity calculation was performed via cosine similarity of the embedded text sections. Newly identified ambiguities, which were resolved by a chemist, were embedded as described above and stored in the CAD.

1.2.2 Priming of *X*DL Database

The *X*DL Database was initialized with 62 procedures-*X*DL pairs that were previously published and experimentally verified[7]. To do so, the procedures were embedded with the model *text-embedding-large* from OpenAI. The embedding alongside the text procedures and corresponding *X*DLs were stored in a vector database. During the generation of new *X*DLs, the current procedure was embedded as described above, and the 5 most similar procedure-*X*DL pairs were extracted (unless otherwise noted) from the *X*DL database and included in the prompt for further *X*DL generation. Successfully translated and validated *X*DLs were added to this database unless otherwise noted.

1.2.3 Structure of Paper Knowledge Graph

During the extraction of synthesis-related data, a knowledge graph (KG) was generated by combining all extracted data. The individually extracted data (see Paper-Scraping-Agent above) were combined into a single object (Figure S18) after each iteration of the Paper-Scraping-Agent.

```
{
    "chemicals": {
        "chemical_name": ["synonyms"],
        "...": ["..."]
    },
    "procedure_titles": [
        "...",
        "..."
    ],
    "procedure_texts": {
        "procedure_title": {
            "full_procedure_text": "...",
            "product": "...",
            "chemicals": [
                "..."
            ],
            "purification": {
                "...": [
                    "conditions"
                ]
            },
            "yield": "...",
            "analysis": {
                "method": [
                    "data"
                ]
            },
            "additional_info": [
                "..."
            ]
        },
        "...": {
            "...": "..."
        }
    }
}
```

Figure S18: Simplified depiction of the KG generated from the extracted data.

1.2.4 *Labbook* structure

A *labbook* was implemented to summarize all data gathered during the translation and validation of a procedure. All chemicals (plus amounts and external data), resolved ambiguities, the initial and sanitized procedure, error-log from the translation, classification,

as well as not executable steps (compare Critique-Agent) were summarized. Additionally, each *labbook* entry is directly mappable to KG and the document database (compare Paper-Scraping-Agent).

1.2.5 Optional Expert Input

Ambiguities that were identified by the Procedure-Agent that the Agent could not resolve itself were optionally answered by an expert chemist. The response alongside the ambiguities was stored alongside previously resolved ambiguities in the CAD. Unless otherwise noted, with module was not used for the conducted experiments.

1.2.6 Filling in Missing Chemical Information

Identified chemical names (Procedure-Agent) were attempted to be unified using the PubChem API and the python package *PubChemPy*. Amounts of identified chemicals in a procedure were unified by converting molar units into mass units. Additionally, a *solvent database* with 1000 commonly used organic chemicals was used to extract and include boiling points, melting points, as well as densities of chemicals[1]. This way, the *X*DL-agent would have the data required to accurately set refluxing temperatures and select the correct phases during a phase separation based upon verified physical data. We considered equipping ACRA with tools to perform a web search of missing data, as shown before[2] but decided against doing so since unverified chemical sources and little control over the extracted data may cause potential safety issues in automatic chemical reaction performance.

1.3 Error Capturing

During the *X*DL generation, generated *X*DLs were verified by first checking for general validity of the *X*DL, then checking for discrepancies between the procedure and the *X*DL, and finally simulating the generated *X*DL in a hardware-constrained environment.

1.3.1 *X*DL-Validity

In the first stage, *X*DLs were validated by checking for syntactic, grammatical, and logical errors. In inspiration of linters as commonly used in modern programming languages, the error messages during this stage always included the error, and the line of the *X*DL procedure causing the error (if possible). A (not exhaustive) overview of the caught errors during this stage is shown in Table S1.

Table S1: Overview of identified errors during the *X*DL Validity stage

Required *X*DL tags are present (opening and closing <XDL>-tags)
All sections required sections are present (Reagent, Hardware, Procedure)
All required tag-attribute are present (e.g. `temp` for the *HeatChill* step)
All unit are in an accepted format (e.g. volume in mL)
All chemicals used in the Procedure section are defined in the Reagent section
All hardware used in the Procedure section are defined in the Hardware section
No duplicate Chemicals are defined
No duplicate Hardware are defined
Only steps implemented in the current version of *X*DL are used
Only attributes implemented for the defined steps are used
StartPurge steps have a corresponding StopPurge step
No unphysical values are given for any units

1.3.2 Discrepancy Check (LLM-as-a-judge)

During the Discrepancy Check stage (also referred to as LLM-as-a-judge[8]) the Critique-Agent (see above) was instructed to identify any missing or wrongly translated steps that do not represent the synthesis procedure. While this stage allows for open-ended decision-making by the Agent it showed to be a valuable addition in the validation of generated procedures (compare main manuscript Figure 4D).

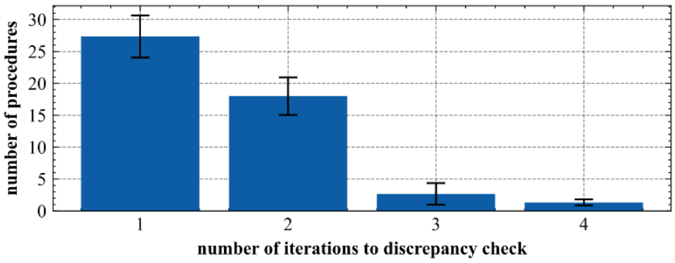

Figure S19: Number of iterations until the discrepancy check stage is reached during the translation process. Three times 50 procedures were translated. Error bars represent the standard deviation of the three runs.

1.3.3 Simulation of Execution

The last stage of the *X*DL validation (simulation of execution) was performed using a predefined hardware graph. Chemicals and Hardware mentioned in the *X*DL were mapped to this hardware graph. The execution was then simulated with the in-built functionality of *X*DL to perform the steps required to perform the synthesis. At this stage hardware hardware-constrained limitations and general executability of the procedures are confirmed (e.g. temperature limits).

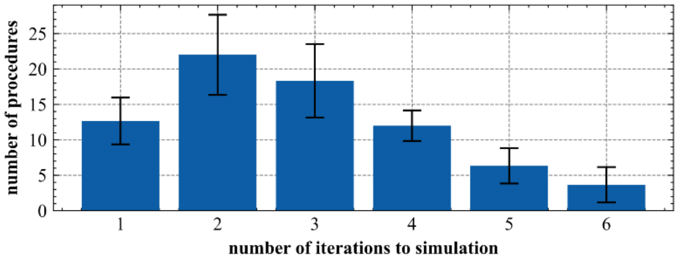

Figure S20: Number of iterations until the simulation stage is reached during the translation process. Three times 50 procedures were translated. Error bars represent the standard deviation of the three runs.

1.3.4 Overall Translation Efficiency

The overall efficiency of the translation of synthesis procedures to *X*DL was evaluated on 150 procedures (three times 50 independently sampled). The maximum number of iterations (*X*DL-generation → Validation → Feedback → *X*DL-generation) for the generation of error-free *X*DL after all three validation stages was set to 6. The distribution of number actual number of iterations is shown in Figure S21.

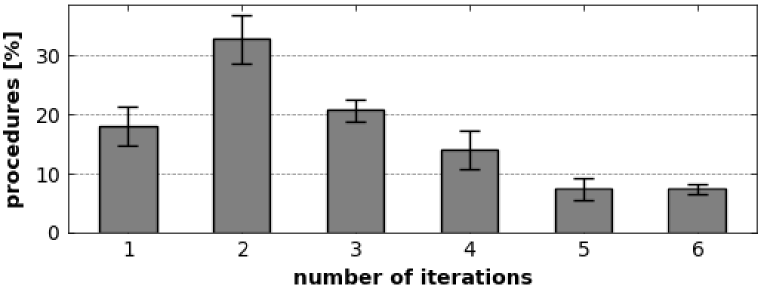

Figure S21: Number of iterations to error-free XDL generation. Three times 50 procedures were translated. Error bars represent the standard deviation of the three runs.

1.4 Parsing of Textual Documents

Similar to prior work, we focused our attention on parsing textual data from literature documents[9,10], though some have extended this to non-textual data[11]. To extract textual information, we used the Python package *pypdf (4.3.1)*. Different from *Bran. et. al.* and others[9,12] we did not preprocess the text documents to filter out relevant sections via rule-based filters, but instead provided the respective Language Model with all textual data extracted from each document.

2 Benchmarks

2.1 Benchmarking the classification of extracted literature procedures by the Procedure Agent

2.1.1 Benchmark Data

For the benchmarking of extracting data from literature procedures, the following 20 manuscripts, along with their supporting information, were used. Only manuscripts published after the 1st of January 2024, were considered to mitigate potential training set contamination.

The automated classification results for the extracted procedures from the first ten literature sources are shown in Figure 3B/C. The results for the latter 10 literature sources are shown in Figure S22.

https://www.nature.com/articles/s41557-024-01528-7

https://www.nature.com/articles/s41557-024-01480-6

https://www.nature.com/articles/s41557-024-01512-1

https://www.nature.com/articles/s41557-024-01504-1

https://www.nature.com/articles/s41557-024-01508-x

https://www.nature.com/articles/s41557-024-01493-1

https://www.nature.com/articles/s41557-024-01506-z

https://www.nature.com/articles/s41557-024-01505-0

https://www.nature.com/articles/s41557-024-01495-z

https://www.nature.com/articles/s41557-024-01481-5

doi.org/10.1002/ejoc.202301230

doi.org/10.1002/ejoc.202300847

doi.org/10.1002/ejoc.202301191

doi.org/10.1002/ejoc.202301028

doi.org/10.1002/ejoc.202300993

doi.org/10.1002/ejoc.202301252

doi.org/10.1002/ejoc.202301262

doi.org/10.1002/ejoc.202301250

doi.org/10.1002/ejoc.202301178

doi.org/10.1002/ejoc.202301001

The following Ph.D. thesis was used to extract data from a thesis-type formatted document.

https://thesis.library.caltech.edu/1058/

For the extraction of data from a non-English document, the following practicum script was used.

https://www.uni-muenster.de/imperia/md/content/organisch_chemisches_institut/pdf/ws2014_2015/organischechemie-grundlagen/grundpraktikum/oc_grund_skript_2015-2016.pdf

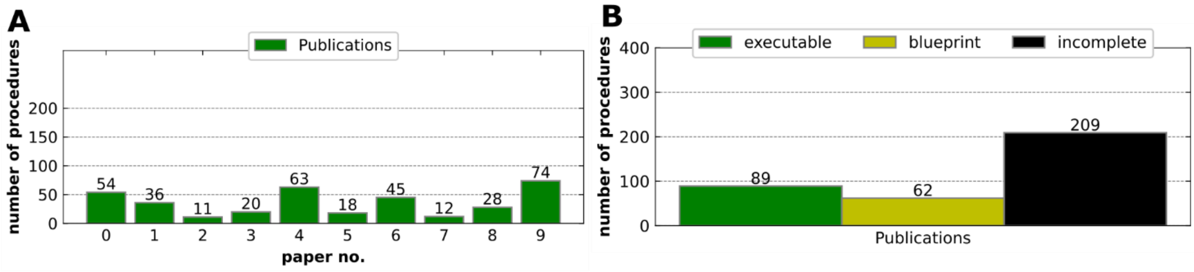

Figure S22: A) Number of extracted procedures from the latter 10 publications listed above. B) Classification of the extracted procedures from A).

2.1.2 Benchmark Results

For the evaluation of the classification of extracted procedures by the Procedure Agent, 39 extracted procedures were randomly selected and manually evaluated. The evaluation was performed relative to the extracted procedure, not the original literature source. The hard-coded classification of procedures as *blueprints* was disabled for this test to solely evaluate the LLMs classification capabilities. Procedures were considered to be correctly classified if:

- **A (executable)**: All chemicals were referenced by uniquely identifiable names (no manuscript-specific abbreviations e.g. Amine 1, the aldehyde, compound A), and all synthetic operations were clearly described.
- **B (blueprint)**: At least one chemical was not referenced by a uniquely identifiable name or consulting additional sources is necessary to perform the synthesis (e.g. reference to a general procedure).
- **C (incomplete)**: All others.

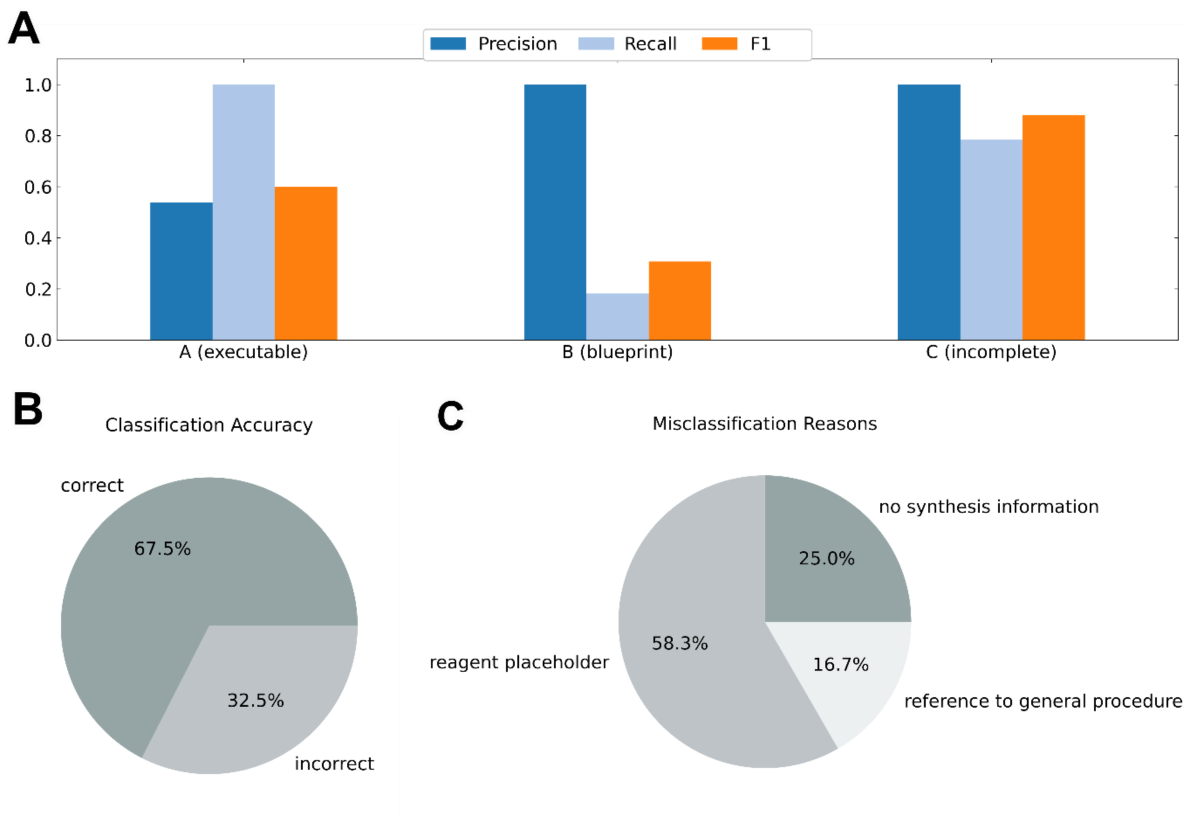

Figure S23: Benchmark results for the classification of extracted procedures by the Procedure Agent. **A/B)** F1, Precision, Recall, and overall accuracy of the classifications. C**)** Misclassification reasons for wrongly classified procedures.

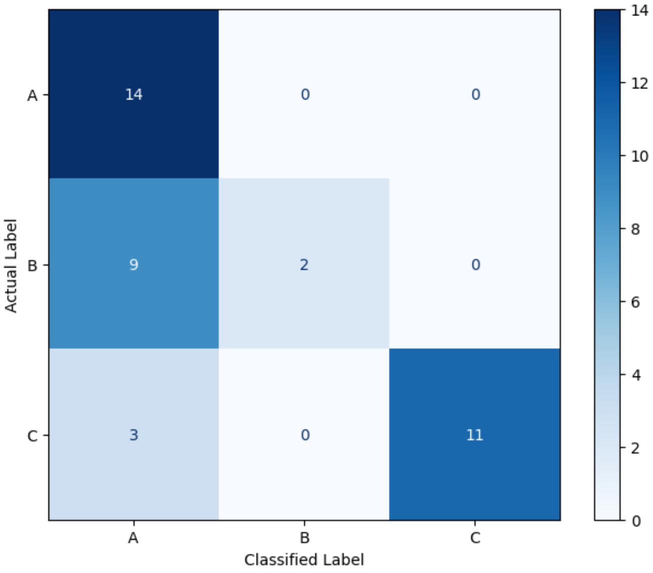

Figure S24: Confusion matrix of classifications benchmark, showing actual label vs classified label.

2.1.3 Sanitization of Literature Procedures

The sanitization quality of extracted procedures was separately evaluated for those written in English and German. For the evaluation of the English procedures, 20 procedures were randomly selected from the previously extracted procedures throughout this study, which were labelled as executable. The evaluation of the German procedures was conducted with 10 randomly sampled procedures from the practicum script mentioned above. All procedures and their sanitized counterparts were manually evaluated for any error outside of the scope of sanitization (e.g. incorrect or swapped steps). The procedures had 0.45 and 0.9 errors per English and German procedure, respectively (Figure S25).

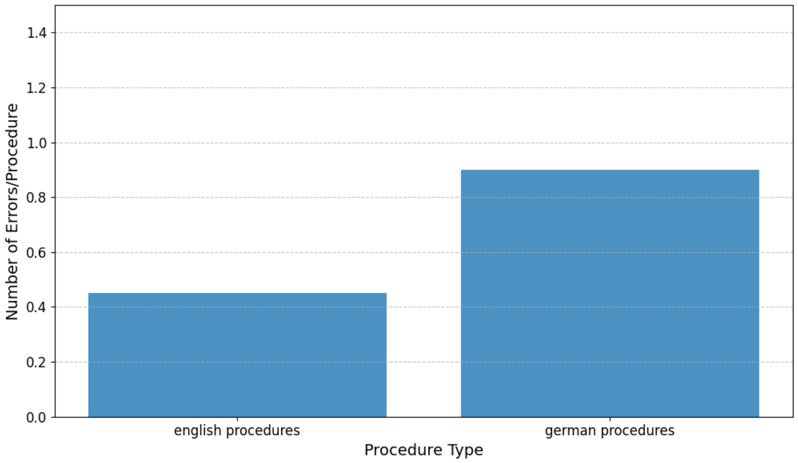

Figure S25: Errors during sanitization of extracted synthesis procedures for procedures written in English and German, respectively.

2.2 Knowledge Graph Extraction

2.2.1 Methodology

Similar to previous approaches, the Paper-Scraping Agent was instructed to extract synthesis-related data in a predefined schema[9,13–16] (compare Figure S4). To validate our approach, we manually confirmed the extraction of analytical data and chemical entities. For the extraction of analytical data from literature sources, 20 of the extracted procedures from the literature sources (section 2.1) were randomly sampled and validated (main manuscript Figure 3E/F). Each extracted value was manually validated. The following analytical data sources were considered, amongst others:

- NMR (1H, 13C, …)
- Mass-Spectrometry
- Retention factors
- UV-vis
- Infrared spectroscopy

For the analysis, duplicate values were classified as false positives (FP). NMR values were considered as correctly extracted with or without multiplicities, or coupling constants (e.g. '4.04' and '4.04 (t, J = 5.9 Hz, 2H)' were both classified as true positive).

For the extraction of chemical entities, the following 8 literature procedures were used as a benchmark set adapted from[17]:

- Org. Synth. 1998, 75, 1 (part A)
- Org. Synth. 1988, 66, 220
- Org. Synth. 1989, 67, 69 (part A)
- Org. Synth. 1989, 67, 69 (part B)
- Org. Synth. 1990, 68, 32 (part A)
- Org. Synth. 1990, 68, 32 (part B)
- Org. Synth. 1990, 68, 206
- Org. Synth. 2009, 86, 194 (part A)

The extraction was validated for direct extraction from the literature source (as commonly used in our pipeline; see Paper-Scraping Agent above) and directly from the synthesis procedure. The benchmark results can be found in Figure 3E in the main manuscript.

2.2.2 Benchmarking ChemDataExtractor 2.0

For comparison ChemDataExtractor 2.0 was evaluated in extracting chemical entities from the same eight synthesis procedures. Chemical entities were considered to be successfully extracted, if the chemical name is part of the extracted entity with or without a prefix such as "glacial" in "(glacial) acetic acid". Duplicated names were only considered once, as long as they could be identified via trivial transformation such as transformation into lower case.

2.3 *X*DL Translation

2.3.1 Methodology

The accuracy of translating synthesis procedures into *X*DL was benchmarked as described in *Mehr et. al.*[17] (Figure 4D main manuscript). For the benchmark, the following settings were considered:

- Direct translation from literature source (the Paper-Scraping Agent first extracts the procedure, which gets subsequently translated into *X*DL; $ACRA_{paper}$ in Figure 4D)
- Translation from the manually extracted procedures ($ACRA_{procedure}$ in Figure 4D)
- Translation from the manually extracted procedures without the Critique Agent ($ACRA_{procedure\text{-}no\text{-}judge}$ in Figure 4D)

2.4 Procedures for Benchmarking Translation Efficiency

The translation efficiency was tested with 150 randomly sampled procedures from the ChemRnD dataset[4]. Three times, 50 independently and randomly sampled procedures were selected and subsequently translated (Table S2).

Table S2: Procedures used for benchmarking translation efficiency of ACRA

exp532.txt	exp22.txt	exp52.txt
exp63.txt	exp480.txt	exp518.txt
exp360.txt	exp283.txt	exp244.txt
exp381.txt	exp65.txt	exp581.txt
exp239.txt	exp27.txt	exp27.txt
exp252.txt	exp116.txt	exp209.txt
exp67.txt	exp268.txt	exp106.txt

exp549.txt	exp77.txt	exp531.txt
exp37.txt	exp358.txt	exp481.txt
exp262.txt	exp47.txt	exp160.txt
exp517.txt	exp553.txt	exp317.txt
exp563.txt	exp427.txt	exp232.txt
exp281.txt	exp50.txt	exp404.txt
exp546.txt	exp429.txt	exp120.txt
exp442.txt	exp229.txt	exp465.txt
exp38.txt	exp578.txt	exp227.txt
exp165.txt	exp534.txt	exp218.txt
exp468.txt	exp122.txt	exp306.txt
exp105.txt	exp58.txt	exp381.txt
exp24.txt	exp275.txt	exp275.txt
exp188.txt	exp310.txt	exp33.txt
exp75.txt	exp172.txt	exp208.txt
exp527.txt	exp495.txt	exp337.txt
exp326.txt	exp280.txt	exp305.txt
exp498.txt	exp489.txt	exp462.txt
exp56.txt	exp462.txt	exp618.txt
exp223.txt	exp328.txt	exp135.txt
exp39.txt	exp367.txt	exp100.txt
exp194.txt	exp238.txt	exp411.txt
exp275.txt	exp455.txt	exp586.txt
exp602.txt	exp464.txt	exp236.txt

exp352.txt	exp205.txt	exp144.txt
exp513.txt	exp366.txt	exp192.txt
exp328.txt	exp23.txt	exp343.txt
exp0.txt	exp316.txt	exp409.txt
exp298.txt	exp151.txt	exp216.txt
exp108.txt	exp163.txt	exp128.txt
exp90.txt	exp128.txt	exp436.txt
exp236.txt	exp590.txt	exp497.txt
exp151.txt	exp499.txt	exp448.txt
exp316.txt	exp60.txt	exp442.txt
exp338.txt	exp390.txt	exp245.txt
exp147.txt	exp25.txt	exp177.txt
exp403.txt	exp203.txt	exp69.txt
exp565.txt	exp502.txt	exp124.txt
exp312.txt	exp575.txt	exp461.txt
exp171.txt	exp565.txt	exp504.txt
exp79.txt	exp331.txt	exp438.txt
exp176.txt	exp431.txt	exp410.txt
exp200.txt	exp359.txt	exp460.txt

2.5 Procedures for the Benchmark of Memory Components

The influence of including the *long-term-memory* (*X*DL-database) and the other memory and data components mentioned above and in the main manuscript for the *X*DL generation in a *few-*

shot prompting style[18] was tested with 75 procedures (three times 25) that were randomly sampled from the ChemRnD database (Table S3; main manuscript Figure 5).

Table S3: Procedure used for the benchmark of the memory components of ACRA

exp453.txt	exp179.txt	exp136.txt
exp466.txt	exp96.txt	exp42.txt
exp135.txt	exp300.txt	exp339.txt
exp337.txt	exp127.txt	exp29.txt
exp57.txt	exp600.txt	exp503.txt
exp546.txt	exp184.txt	exp407.txt
exp471.txt	exp488.txt	exp465.txt
exp378.txt	exp192.txt	exp389.txt
exp538.txt	exp122.txt	exp576.txt
exp428.txt	exp312.txt	exp251.txt
exp79.txt	exp537.txt	exp580.txt
exp486.txt	exp281.txt	exp451.txt
exp563.txt	exp303.txt	exp536.txt
exp226.txt	exp125.txt	exp537.txt
exp358.txt	exp200.txt	exp432.txt
exp227.txt	exp120.txt	exp98.txt
exp186.txt	exp564.txt	exp524.txt
exp329.txt	exp323.txt	exp340.txt
exp59.txt	exp615.txt	exp22.txt
exp96.txt	exp226.txt	exp280.txt
exp233.txt	exp511.txt	exp68.txt

exp384.txt	exp85.txt	exp406.txt
exp19.txt	exp84.txt	exp542.txt
exp166.txt	exp485.txt	exp45.txt
exp402.txt	exp351.txt	exp471.txt

2.6 Output comparison of different models

We tested the potential use of smaller language models for the translation of synthetic procedures into *X*DL using two state-of-the-art open-source models. We chose one *reasoning-model* (*deekseek-r1 70b*[19]) and one pretrained model (*llama 3.3 70b*[20]) for our evaluation. Both models were set up and instructed using *ollama*[21] (Figures S25 and S26, gpt-4o in comparison in Figure S27) with the same prompt for *X*DL generation as used throughout the rest of our work (SI section 1.1.4). Neither of the models generated valid *X*DLs in our test, showing the necessity of extended finetuning for competitive performance of smaller models for this task.

```
<Procedure>
  <Add substance="N,N-Dimethylformamide" amount="0.1μL" unit="volume"/>
  <Add substance="4-(N,N-Dimethylamino)benzaldehyde" amount="Not Specified"/>
  <Add substance="Nitromethane" amount="Not Specified"/>
  <Add substance="Piperidine" amount="Not Specified"/>
  <Mix substance1="N,N-Dimethylformamide" ratio1="9" substance2="Methanol"
ratio2="1"/>
</Procedure>
```

Figure S26: Exemplary output from a synthetic procedure to XDL translation using llama 3.3 70b.

```xml
<XDL>
  <!-- Step 1: Extract Chemicals -->
  <extract_chemicals>
    {
      "N,N-dimethylformamide": {
        "role": "solvent",
        "amount": "0.1 mL"
      },
      "4-(dimethylamino)benzaldehyde": {
        "role": "substrate",
        "amount": "1 g"
      },
      "tosyl chloride": {
        "role": "activating-agent",
        "amount": "0.15 g"
      },
      "pyridine": {
        "role": "base",
        "amount": "0.12 mL"
      }
    }
  </extract_chemicals>

  <!-- Step 2: Prepare Solution of Tosyl Chloride in DMF -->
  <prepare_solution>
    <reactor>reactor_1</reactor>
    <components>
      <component>
        <chemical>tosyl chloride</chemical>
        <amount>0.15 g</amount>
        <unit>g</unit>
      </component>
      <component>
        <chemical>N,N-dimethylformamide</chemical>
        <amount>5 mL</amount>
        <unit>mL</unit>
      </component>
    </components>
  </prepare_solution>

  <!-- Step 3: Transfer Tosyl Chloride Solution to Main Reactor -->
  <transfer>
    <source_reactor>reactor_1</source_reactor>
    <target_reactor>main-reactor</target_reactor>
    <volume>5 mL</volume>
  </transfer>

  <!-- Step 4: Add Substrate to Main Reactor -->
  <add>
    <reactor>main-reactor</reactor>
    <components>
      <component>
        <chemical>4-(dimethylamino)benzaldehyde</chemical>
        <amount>1 g</amount>
        <unit>g</unit>
      </component>
    </components>
  </add>

  <!-- Step 5: Add Pyridine to Main Reactor -->
  <add>
    <reactor>main-reactor</reactor>
    <components>
      <component>
        <chemical>pyridine</chemical>
        <amount>0.12 mL</amount>
        <unit>mL</unit>
      </component>
    </components>
  </add>

  <!-- Step 6: Stir the Reaction Mixture -->
  <stir>
    <reactor>main-reactor</reactor>
    <duration>30 min</duration>
  </stir>
</XDL>
```

Figure S27: Exemplary output from a synthetic procedure to XDL translation using deepseek-r1 70b.

```xml
<XDL>
    <Synthesis>
        <Hardware>
            <Component id="reactor"
                       type="reactor" />
            <Component id="rotavap"
                       type="rotavap" />
            <Component id="cartridge_basic_aluminum_oxide"
                       type="cartridge"
                       chemical="basic aluminum oxide" />
        </Hardware>
        <Reagents>
            <Reagent name="4-{[2-(4-carboxy-phenyl)-pyrimidine-5-carbonyl]-cyclopropyl-amino}-piperidine-1-carboxylic
acid tert-butyl ester"
                     role="reagent"
                     solid="True" />
            <Reagent name="N,N-dimethylformamide"
                     role="solvent" />
            <Reagent name="ethyldiisopropylamine"
                     role="base" />
            <Reagent name="2-(1H-benzotriazol-1-yl)-1,1,3,3-tetramethyluronium tetrafluoroborate"
                     role="activating-agent"
                     solid="True" />
            <Reagent name="corresponding amine"
                     role="reagent" />
            <Reagent name="aqueous K2CO3 solution (3 M)"
                     role="base" />
            <Reagent name="N,N-dimethylformamide/methanol (9:1)"
                     role="solvent" />
        </Reagents>
        <Procedure>
            <Add vessel="reactor"
                 reagent="4-{[2-(4-carboxy-phenyl)-pyrimidine-5-carbonyl]-cyclopropyl-amino}-piperidine-1-carboxylic
acid tert-butyl ester"
                 amount="4.7 mg" />
            <Add vessel="reactor"
                 reagent="N,N-dimethylformamide"
                 volume="0.1 uL"
                 stir="True" />
            <Stir vessel="reactor"
                  time="10 min"
                  stir_speed="300" />
            <Add vessel="reactor"
                 reagent="ethyldiisopropylamine"
                 volume="5 uL" />
            <Add vessel="reactor"
                 reagent="2-(1H-benzotriazol-1-yl)-1,1,3,3-tetramethyluronium tetrafluoroborate"
                 amount="3.2 mg" />
            <HeatChill vessel="reactor"
                       temp="25"
                       time="10 min"
                       stir="True"
                       stir_speed="300" />
            <Add vessel="reactor"
                 reagent="corresponding amine"
                 amount="10 umol" />
            <HeatChill vessel="reactor"
                       temp="25"
                       time="12 h"
                       stir="True"
                       stir_speed="300" />
            <Add vessel="reactor"
                 reagent="aqueous K2CO3 solution (3 M)"
                 volume="20 uL" />
            <Stir vessel="reactor"
                  time="10 min"
                  stir_speed="300" />
            <FilterThrough from_vessel="reactor"
                           to_vessel="rotavap"
                           through="basic aluminum oxide" />
            <WashSolid vessel="rotavap"
                       solvent="N,N-dimethylformamide/methanol (9:1)"
                       volume="sufficient" />
            <Evaporate vessel="rotavap"
                       mode="auto" />
        </Procedure>
    </Synthesis>
</XDL>
```

Figure S28: Exemplary output from a synthetic procedure to XDL translation using gpt-4o.

3. Comparison to previous projects

In previous studies, semantic parsing was used to translate literature procedures into *X*DL. While this proved to be a powerful tool for further automation of synthetic chemistry, our approach greatly improves the automation of the previously proposed workflow. Notably, we not only allow starting from a given literature procedure, but from the raw literature source, allowing us to include other relevant data from the literature source as well as directly associating analytical data if available. Additionally, we automate many of the previously still manual steps (for instance, converting units). Importantly, we also include validating that the procedure is correctly represented in the *X*DL and validating its executability in a realistic setup. Notably, our approach has the great advantage of being flexible to version changes of *X*DL and allows us to include previous experience to increase the success rate of translating procedures.

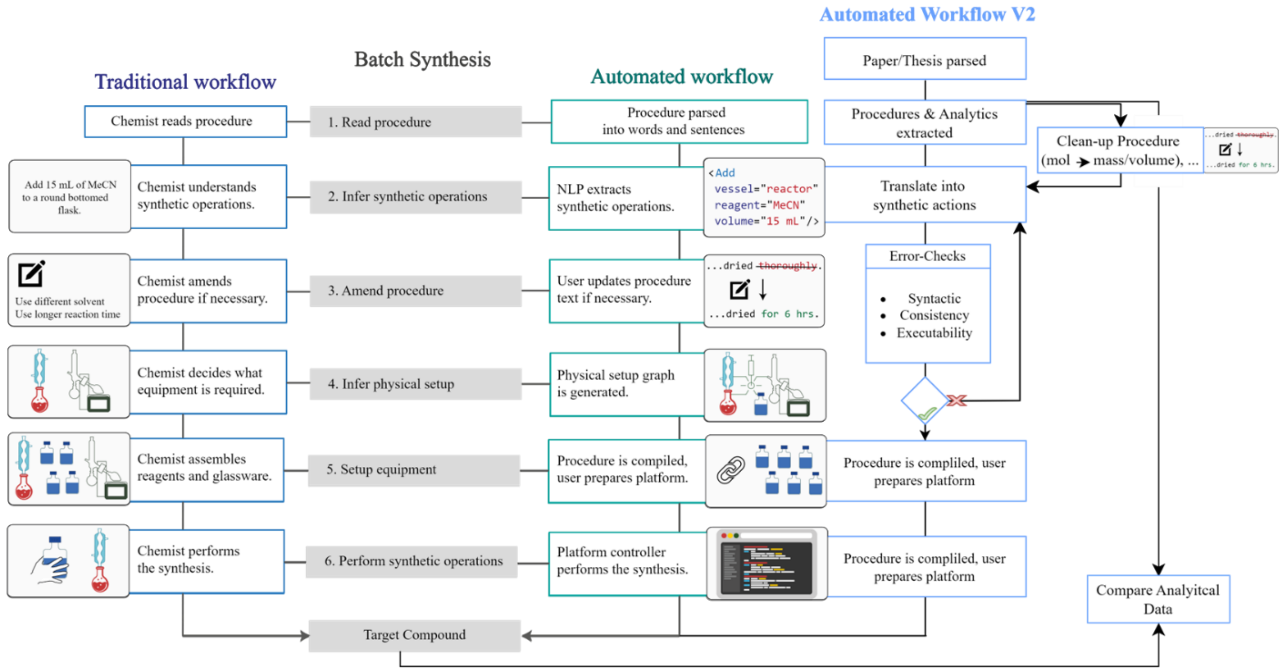

Figure S29: Comparison of ACRA to previously reported SynthReader[17] (Figure adapted and edited from literature).

4 Reaxys, ORD, and analysis, not executable steps

To suggest a roadmap for truly and continuously universal *X*DL we compared keywords provided with the Reaxys database[22] as the abstract reaction steps, n-grams extracted from procedures of the Open Reaction Database[10] (ORD), and steps identified as not executable by the Critique-Agent.

4.1 Procedure Keyword Analysis from the Reaxys Database

The 200 most frequently appearing keywords among the Reaxys Database of synthetic procedures were manually clustered into 20 categories (Figure S29). Additionally, the 1000 most frequent keywords were manually checked for any other potential clusters, but no further clusters could be identified. Out of the 20 identified clusters, 9 were categorized as currently not implemented in *X*DL.

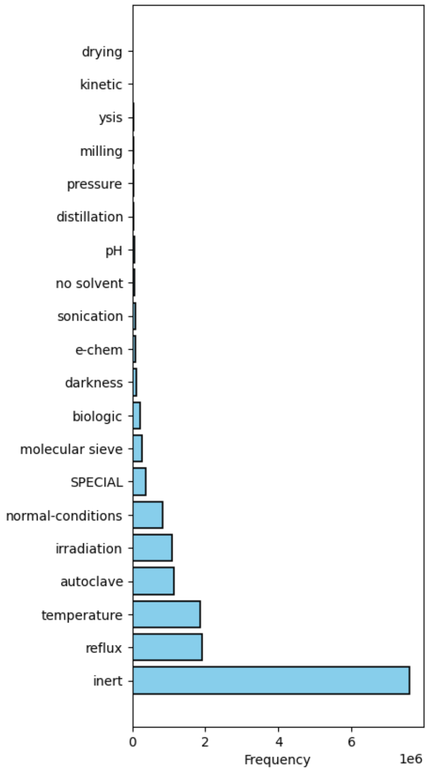

Figure S30: Category frequencies the 200 most frequently used keywords provided in the Reaxys Database were clustered into.

4.2 Most frequent n-grams in the Open Reaction Database

Additionally, to the clusters identified above, the procedures provided in the ORD were analysed by identifying the 200 most frequent n-grams[23] (where n=3). Similarly to above the resulting tri-grams were clustered, yielding 12 unique categories (Figure S30). Out of these only two were identified as not implemented in the *X*DL version in this study (NMR and Chromatography).

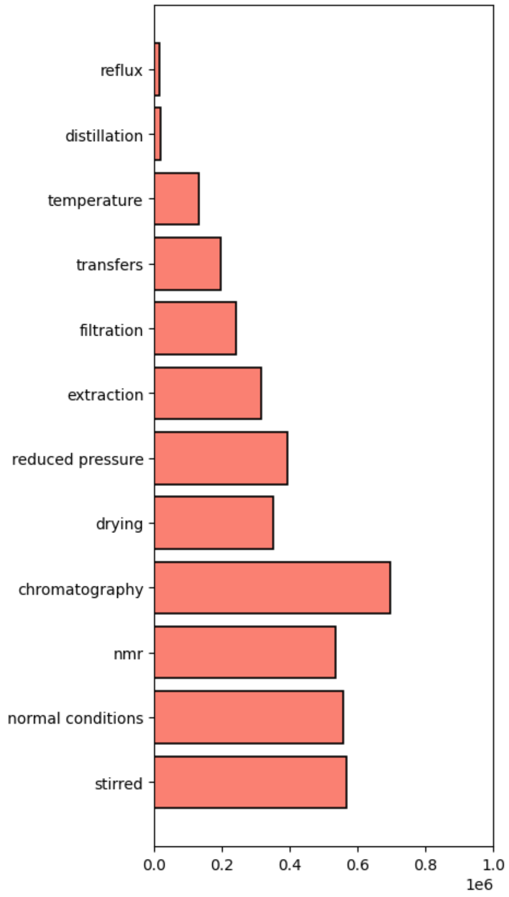

Figure S31: Category frequency of the 200 most frequent tri-grams in the ORD were clustered into.

4.3 Analysis of not executable steps

To identify new process steps from those marked as non-executable by the Critique-Agent, a total of 350 steps identified during this study were manually analyzed and clustered to generate new step suggestions. Among the 26 new suggestions, the proposed modifications range from

introducing new attributes for existing process steps (e.g., specifying the rate of heating for *HeatChill* steps) to incorporating steps that necessitate additional hardware support (e.g., weighing or grinding). Notably, the analysis of steps marked as non-executable yielded more detailed recommendations, as illustrated in Figure S31.

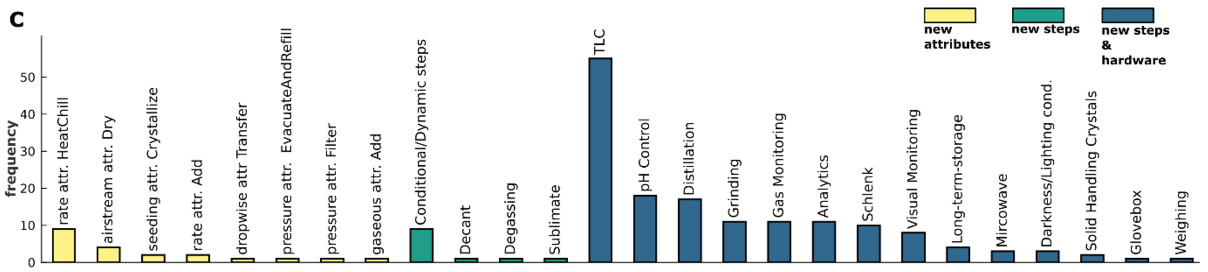

Figure S32: 26 new feature suggestions for the future development of XDL after the analysis of as not executable marked synthetic steps by the Critique-Agent.

This analysis furthermore proved to be of substantial importance, since a large percentage of translated procedures proved to have non-executable steps as identified by the Critique-Agent (Figure S32).

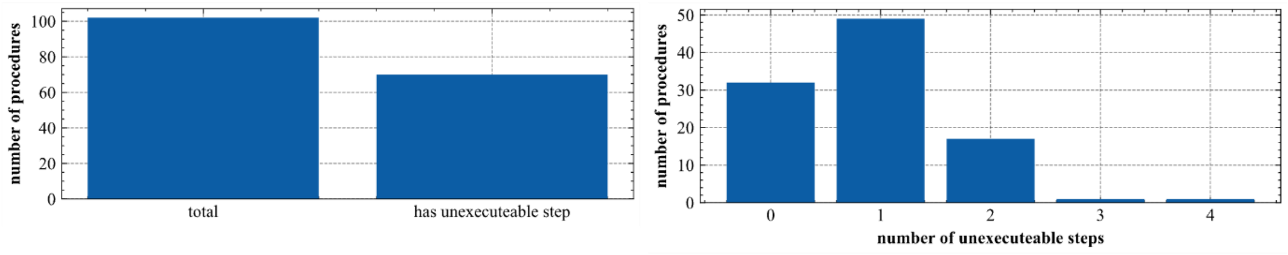

Figure S33: Left) Number of procedures (out of 100) for which the Critique-Agent identified not executable steps. **Right)** Distribution of non-executable steps per procedure after 100 translations.

5. Details of performed synthesis

5.1 Hardware implementation

The notebooks of synthesis execution, graphs, XDL files and log files are in Zenodo under the section "synthesis".

5.1.1 Chemputer

The Chemputer platform used to perform automated syntheses without human intervention was built following previously published hardware configurations.[7,17,24,25] The liquid handling backbone consisted of four syringe pumps and four six-way valves forming the primary fluidic backbone. The syringe pumps provide precise volumetric control for aspirating and dispensing solutions throughout the system. Each pump connects to a six-way valve, enabling routing of liquids to multiple destinations. An additional four daisy-chained valves extend the connectivity of the backbone, providing sufficient ports for all input solution bottles, reactor vessels, and processing modules required for the syntheses described in this work. Further, the platform was equipped with an automated separation funnel, enabling liquid-liquid extraction workups. A conductivity sensor integrated into the separation funnel detects the interface between aqueous and organic phases, allowing automated layer separation without human intervention. A drying cartridge packed with anhydrous magnesium sulfate ($MgSO_4$) provides automated drying of organic solutions following aqueous workup. Solutions are passed through the cartridge via the backbone pumps, with the drying agent removing residual water before subsequent processing steps. Chemputer was further equipped with a hot plate stirrer providing heating and stirring and reactors with air-cooled reflux condensers. A rotary evaporator was integrated into the Chemputer and transfer of solutions into and out of the rotary evaporator flask is accomplished via tubing connected to the backbone pumps and valves. Multiple bottles containing stock solutions of reagents, solvents, and starting materials connect to the backbone

via the daisy-chained valve network. Each bottle is assigned to a specific valve port position recorded in the hardware graph file. Inert gas lines provide positive pressure to bottles containing air-sensitive reagents when required (**Figure S34**).

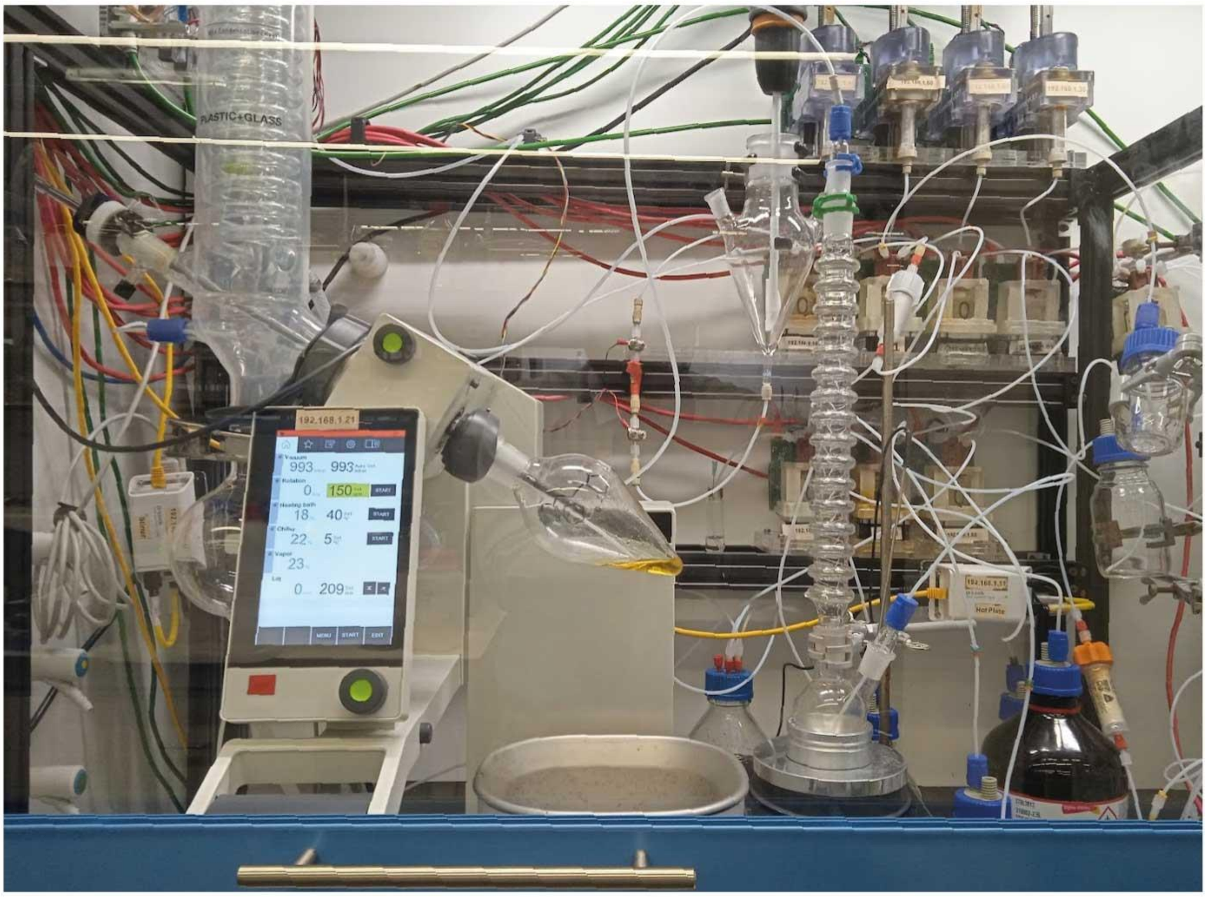

Figure S34: Chemputer with 4 pumps and 4 valves as a basic backbone, with additional 4 daisy-chained valves, rotatory evaporator, $MgSO_4$ cartridge, automated separation funnel and input solution bottles.

5.1.2 Opentrons OT2

As previously described,[25] the Opentrons OT-2 liquid handling robot was integrated with the XDL framework via the OpentronsXDL software layer. This enabled execution of XDL procedures on the commercial pipetting platform. The Opentrons OT-2 was equipped with 8 mL vials holder for stock solutions positioned on the OT-2 deck. A BioShake 3000-T (QInstruments) was integrated with the OT-2 platform to provide temperature control and orbital shaking for reactions. The OpentronsXDL software interfaces with the BioShake controller to execute temperature and shaking commands specified in XDL procedures.

Temperature ramps, hold times, and shaking speeds are controlled programmatically according to XDL step parameters. Operations requiring hardware not available on the OT-2 platform (rotary evaporation, liquid-liquid separation, filtration, drying) are not included in the Opentrons XDL instruction set. Procedures translated for Opentrons execution were therefore limited to reactions where crude product isolation is acceptable or where minimal workup is required.

5.1 Synthesis of 3-Methoxy-3-oxopropanoic acid

Original Procedure:

In einem Reaktionskolben mit Rückflusskühler werden 12.5 g (125 mmol)Bernsteinsäureanhydrid in 6.1 mL (150 mmol, 1.2 Äq.) absolutem Methanol suspendiert und unter Rückfluss erhitzt, bis eine homogene Lösung entstanden ist (ca. 15–30 Minuten). Daraufhin wird das Heizbad so weit abgesenkt, dass das Reaktionsgemisch im Kolben nur noch etwa zur Hälfte ins Heizbad eintaucht, und das Gemisch weitere 20 Minuten gerührt. Nach Abkühlen wird das Methanol am Rotationsverdampfer unter vermindertem Druck entfernt. Der ölige Rückstand wird unter kräftigem Rühren mit dem Spatel im Wasserbad abgekühlt und der so erhaltene Rückstand im Ölpumpenvakuum getrocknet.

Sanitized Procedure:

In a reaction flask equipped with a reflux condenser, suspend 12.5 g (125 mmol) of succinic anhydride in 6.1 mL (150 mmol, 1.2 equivalents) of absolute methanol. Heat the mixture under reflux until a homogeneous solution is formed (approximately 15–30 minutes). Lower the heating bath so that the reaction mixture in the flask is only half-submerged in the heating bath, and stir the mixture for an additional 20 minutes. After cooling, remove the methanol under reduced pressure using a rotary evaporator. Cool the oily residue in a water bath while stirring vigorously with a spatula, and then dry the residue under vacuum using an oil pump.

```xml
<XDL>
  <Synthesis>
    <Hardware>
      <Component id="reactor" type="reactor" />
      <Component id="rotavap" type="rotavap" />
    </Hardware>
    <Reagents>
      <Reagent name="succinic anhydride" role="reagent" solid="True" />
      <Reagent name="absolute methanol" role="solvent" />
    </Reagents>
    <Procedure>
      <Add vessel="reactor" reagent="succinic anhydride" amount="12.5 g" />
      <Add vessel="reactor" reagent="absolute methanol" volume="6.1 mL" />
      <HeatChill vessel="reactor" temp="65 °C" time="30 min" stir="True" stir_speed="300" />
      <HeatChill vessel="reactor" temp="50 °C" time="20 min" stir="True" stir_speed="300" />
      <HeatChillToTemp vessel="reactor" temp="25 °C" />
      <Transfer from_vessel="reactor" to_vessel="rotavap" volume="all" />
      <Evaporate vessel="rotavap" mode="auto" />
      <HeatChillToTemp vessel="rotavap" temp="25 °C" stir="True" stir_speed="280" />
      <Dry vessel="rotavap" time="1 h" pressure="10 mbar" />
    </Procedure>
  </Synthesis>
</XDL>
```

Figure S35: Automatically translated XDL procedure of 3-methoxy-3-oxopropanoic acid.

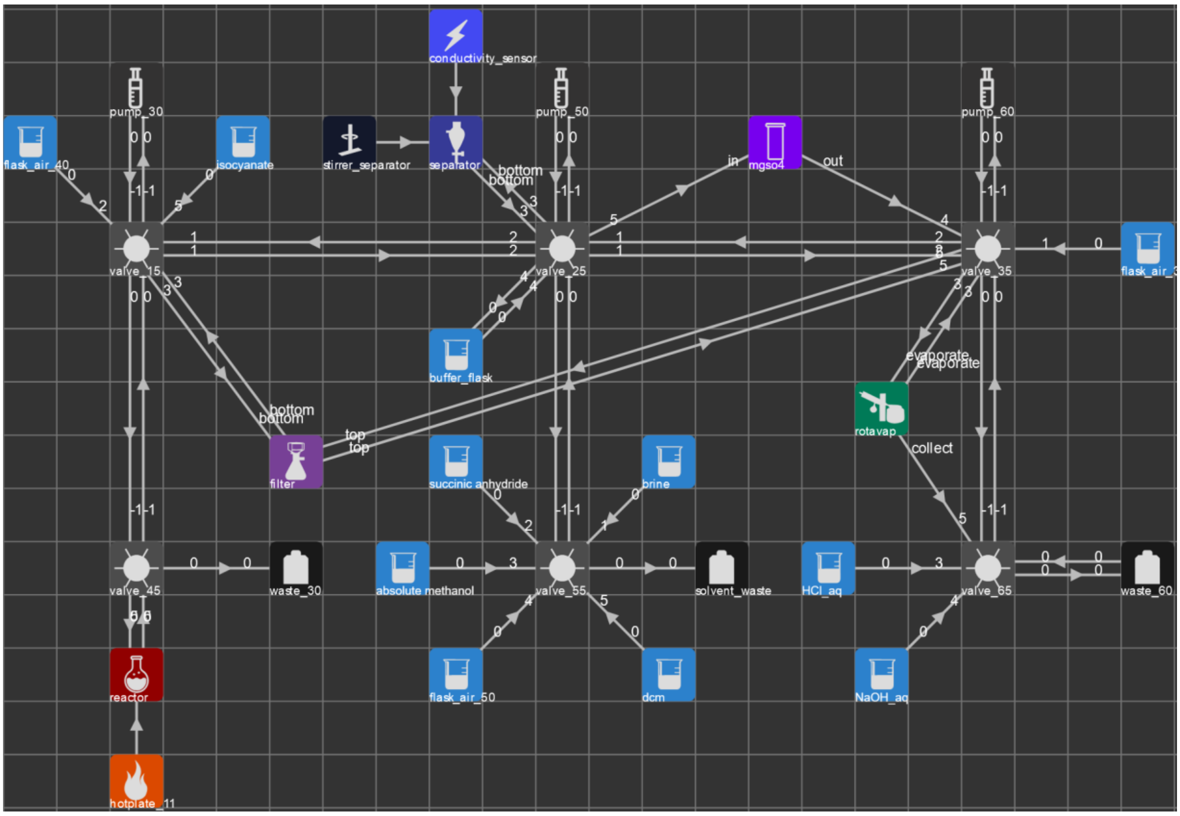

Figure S36: Chemputer graph for procedure of 3-methoxy-3-oxopropanoic acid.

The product was found in rotatory evaporated as white solid, 13.841 g, 84% yield.

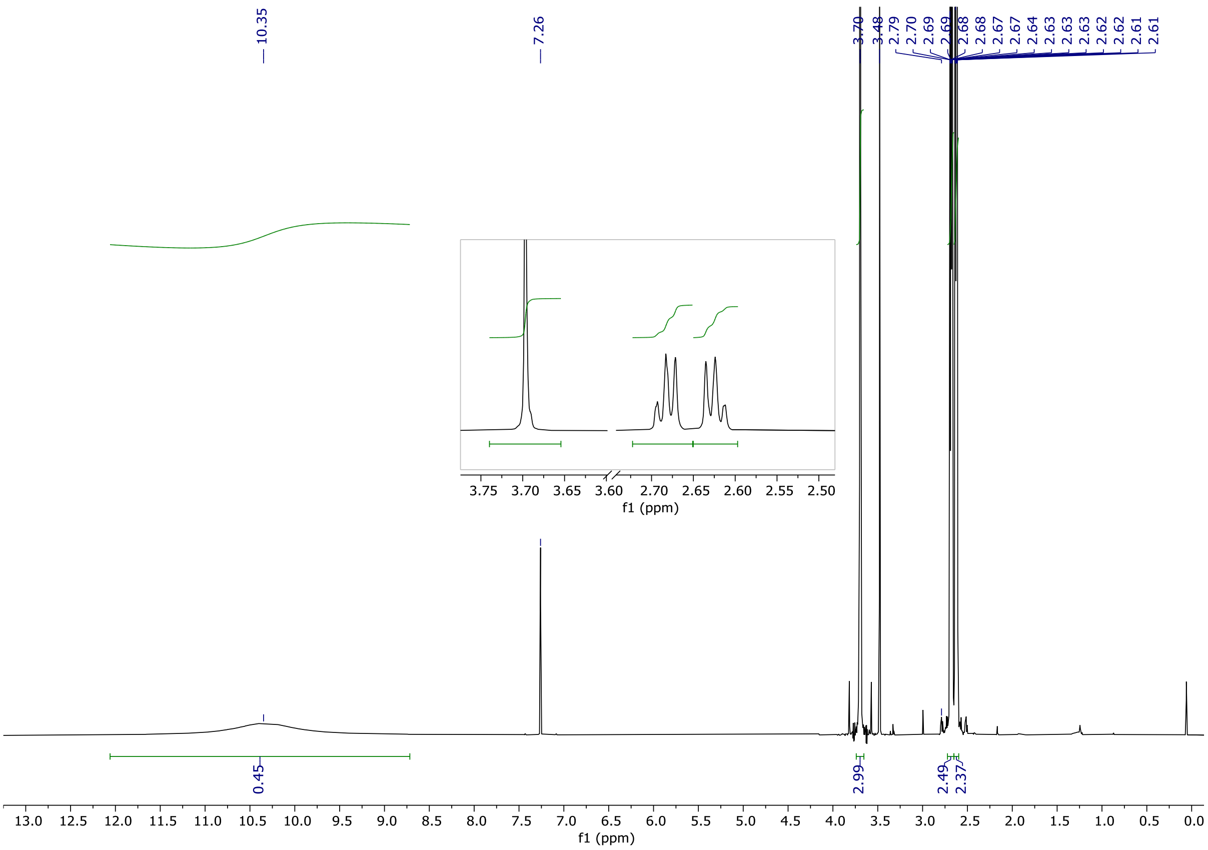

Figure S37: 1H NMR 600 MHz of product 3-methoxy-3-oxopropanoic acid.

5.2 Synthesis of p-toluenesulfonate

Original Procedure:

Under Ar atmosphere, a 100 mL round bottom flask was charged with 1-naphtol (2 g, 13.89 mmol, 1 equiv), DMAP (0.170 g, 1.389 mmol, 0.1 equiv), Et 3N (2.13 mL, 15.28 mmol, 1.1 equiv) and anhydrous CH 2Cl 2 (50 mL). Then, a solution of tosylchloride (2.91 g, 15.28 mmol, 1.1 equiv) in anhydrous CH 2Cl 2 (10 mL) was added dropwise via cannula. The resulting solution was stirred during 15 min. Then, the reaction mixture was transferred to a separatory funnel and NaHSO4 (40 mL, 0.5 M) was added. The layers were separated and the aqueous layer was washed with CH2 Cl 2 (3 × 20 mL) and then with brine (20 mL). The organic phase was dried over MgSO4 and concentrated under reduced pressure. The crude residue was purified by flash chromatography (hexane/EtOAc: 3/1) to obtain 4.05 g of compound 2ae (98% yield).

Sanitized Procedure:

Under an argon atmosphere, a 100 mL round bottom flask was charged with 1-naphthol (2 g, 13.89 mmol, 1 equiv), DMAP (0.170 g, 1.389 mmol, 0.1 equiv), triethylamine (Et3N) (2.13 mL, 15.28 mmol, 1.1 equiv), and anhydrous dichloromethane (CH2Cl2) (50 mL). Then, a solution of tosyl chloride (2.91 g, 15.28 mmol, 1.1 equiv) in anhydrous CH2Cl2 (10 mL) was added dropwise via cannula. The resulting solution was stirred for 15 minutes. Subsequently, the reaction mixture was transferred to a separatory funnel, and 0.5 M sodium bisulfate (NaHSO4) solution (40 mL) was added. The layers were separated, and the aqueous layer was washed with CH2Cl2 (3 *20 mL) and then with brine (20 mL). The organic phase was dried over magnesium sulfate (MgSO4) and concentrated under reduced pressure. The crude residue was purified by flash chromatography using a hexane/ethyl acetate (3/1) mixture to obtain 4.05 g of compound 2ae (98% yield).

Note: Due to hardware limitations, the flash chromatography was omitted for the translation into XDL.

```
<XDL>
<Synthesis>
    <Hardware>
        <Component id="reactor" type="reactor" />
        <Component id="flask" type="flask" />
        <Component id="separator" type="separator" />
        <Component id="flask_organic" type="flask" />
        <Component id="flask_aqueous" type="flask" />
        <Component id="flask_dried" type="flask" />
        <Component id="rotavap" type="rotavap" />
        <Component id="cartridge_MgSO4" type="cartridge" chemical="MgSO4" />
        <!-- <Component id="flask_product" type="flask" /> -->
    </Hardware>
    <Reagents>
        <Reagent name="1-naphthol" role="reagent" solid="True" />
        <Reagent name="DMAP" role="catalyst" solid="True" />
        <Reagent name="Et3N" role="base" />
        <Reagent name="CH2Cl2" role="solvent" />
        <Reagent name="tosyl chloride" role="reagent" solid="True" />
        <Reagent name="NaHSO4" role="acid" />
        <Reagent name="brine" role="reagent" />
        <Reagent name="MgSO4" role="drying agent" />
        <Reagent name="hexane" role="solvent" />
        <Reagent name="EtOAc" role="solvent" />
    </Reagents>
    <Procedure>
        <EvacuateAndRefill vessel="reactor" gas="argon" repeats="2" />
        <Add vessel="reactor" reagent="1-naphthol" amount="2 g" stir="True" stir_speed="300" />
        <Add vessel="reactor" reagent="DMAP" amount="0.170 g" stir="True" stir_speed="300" />
        <Add vessel="reactor" reagent="Et3N" volume="2.13 mL" />
        <Add vessel="reactor" reagent="CH2Cl2" volume="50 mL" stir="True" stir_speed="300" />
        <Add vessel="flask" reagent="tosyl chloride" amount="2.91 g" />
        <Add vessel="flask" reagent="CH2Cl2" volume="10 mL" stir="True" stir_speed="300" />
        <Transfer from_vessel="flask" to_vessel="reactor" volume="all" time="10 min" viscous="True" />
        <Stir vessel="reactor" time="15 min" stir_speed="300" />
        <Transfer from_vessel="reactor" to_vessel="separator" volume="all" />
        <Add vessel="separator" reagent="NaHSO4" volume="40 mL" />
        <Separate purpose="extract" from_vessel="separator" separation_vessel="separator" to_vessel="f
lask_organic"
product_phase="bottom" waste_phase_to_vessel="flask_aqueous" />
        <Repeat repeats="3">
<Transfer from_vessel="flask_aqueous" to_vessel="separator" volume="all" />
            <Separate purpose="wash" from_vessel="separator" separation_vessel="separator"
to_vessel="flask_organic" waste_phase_to_vessel="flask_aqueous" solvent="CH2Cl2" solvent_volume="20 m
L"
product_phase="bottom" />
        </Repeat>
        <Transfer from_vessel="flask_organic" to_vessel="separator" volume="all" />
        <Separate purpose="wash" from_vessel="separator" separation_vessel="separator" to_vessel="flas
k_organic"
waste_phase_to_vessel="flask_aqueous" solvent="brine" solvent_volume="20 mL" product_phase="bottom" />
        <FilterThrough from_vessel="flask_organic" to_vessel="flask_dried" through="MgSO4" />
        <Transfer from_vessel="flask_dried" to_vessel="rotavap" volume="all" />
        <Evaporate vessel="rotavap" mode="auto" />
    </Procedure>
</Synthesis>
</XDL>
```

Figure S38: Automatically translated XDL procedure of tosyl-napthan-1-ol.

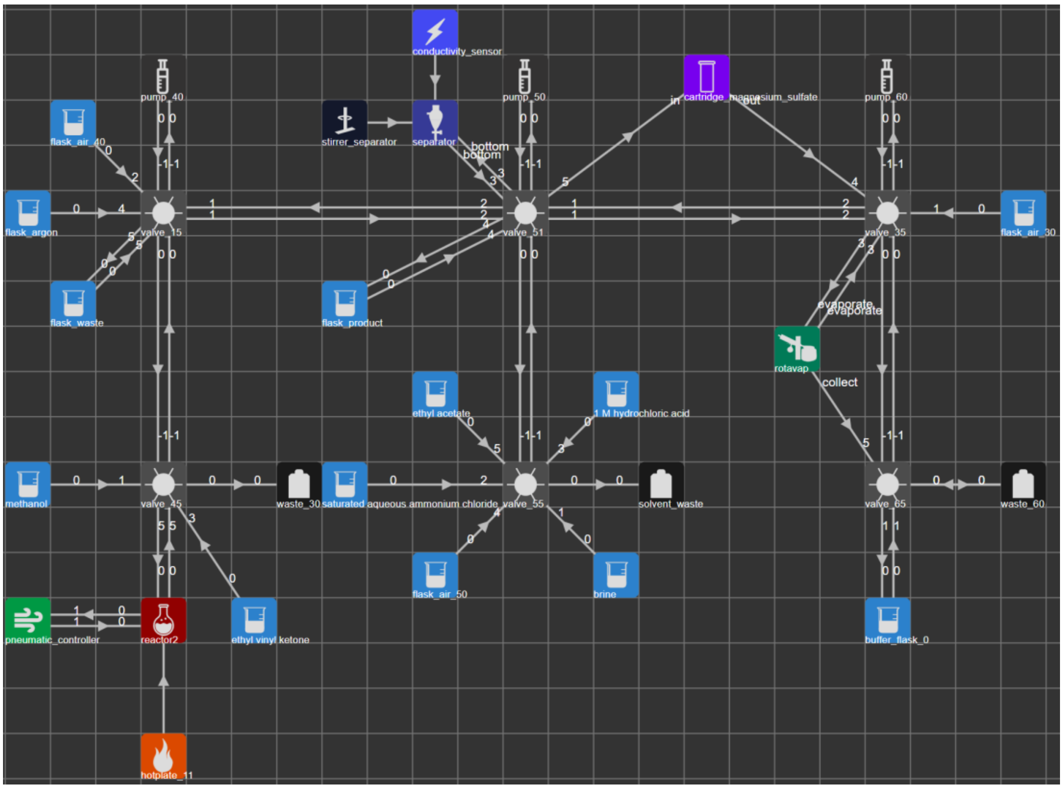

Figure S39: *Chemputer graph for procedure of tosyl-napthan-1-ol.*

The product was found in the rotatory evaporator as a dark oil, 3.32g, 82% yield.

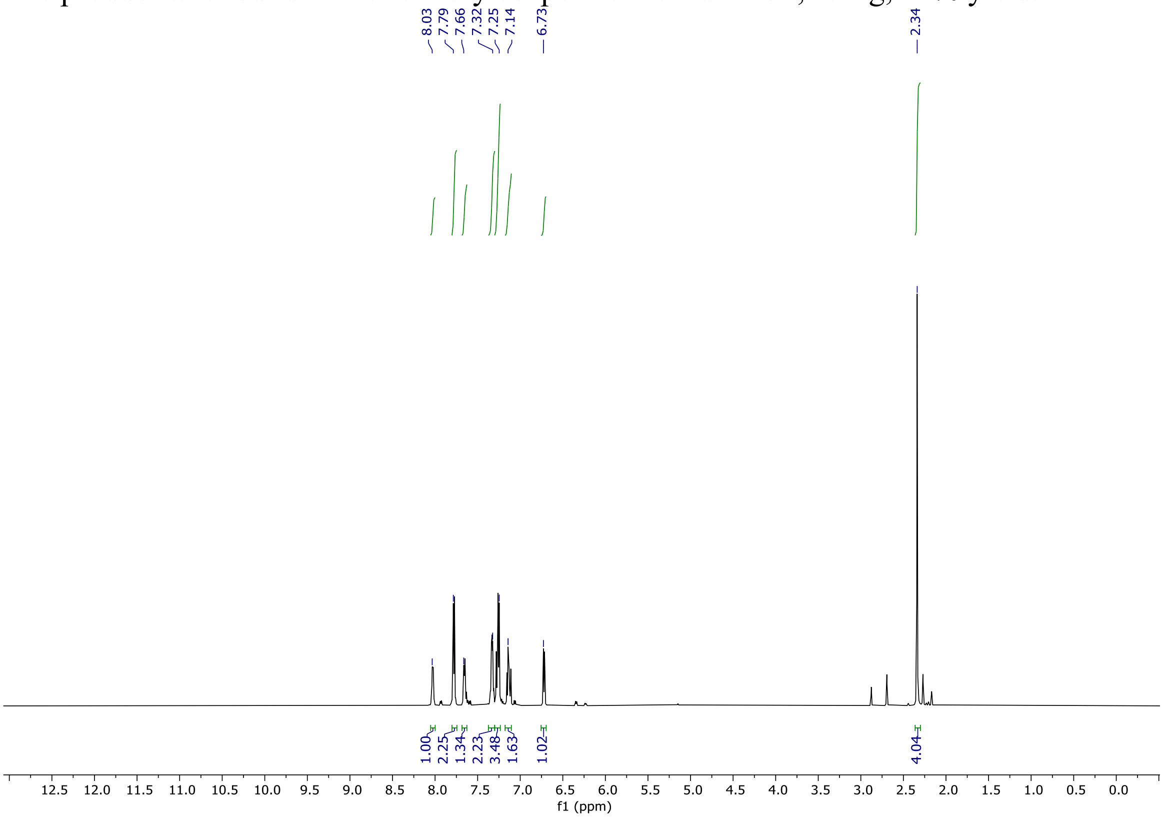

Figure S40: 1H NMR 600 MHz of product tosyl-napthan-1-ol.

5.3 Synthesis of 2-Methyl-2-(3-oxopentyl)-1,3-cyclohexanedione

Original Procedure:

To a refluxing solution of 5.0 g (39.6 mmol) of 2-methyl-1,3-cyclohexanedione and 89 mg (1.6 mmol) of potassium hydroxide in 30 mL of dry methanol under argon, was added 5.0 g (59.4 mmol) of ethyl vinyl ketone over 5 min. After refluxing for 12 h, the solution was diluted with saturated aqueous ammonium chloride and 1 M hydrochloric acid to pH ~ 7.0, and extracted with ethyl acetate three times. The combined extracts were washed with brine, dried (MgSO4), and concentrated to give 8.32 g (100% yield) of 2-methyl-2-(3-oxopentyl)cyclohexane-1,3 dione as a yellow oil, which was used in the next reaction without purification.

Sanitized Procedure:

To a refluxing solution of 5.0 g (39.6 mmol) of 2-methyl-1,3-cyclohexanedione and 89 mg (1.6 mmol) of potassium hydroxide in 30 mL of dry methanol under argon, add 5.0 g (59.4 mmol) of ethyl vinyl ketone over 5 minutes while stirring at 300 rpm. Continue refluxing the mixture at the boiling point of methanol (64.7°C) while stirring at 300 rpm for 12 hours. After 12 hours, cool the solution to room temperature (25°C) and add saturated aqueous ammonium chloride and 1 M hydrochloric acid while stirring at 300 rpm until the pH reaches exactly 7.0. Extract the solution with 30 mL of ethyl acetate three times. Combine the extracts and wash with 30 mL of brine. Dry the combined extracts using magnesium sulfate (MgSO4) by adding it to the solution and stirring at 300 rpm for 10 minutes. Filter the solution to remove the drying agent and concentrate the filtrate under reduced pressure (>100 mbar, temp. 30°C) to obtain 8.32 g of 2-methyl-2-(3-oxopentyl)cyclohexane-1,3-dione as a yellow oil.

```
<XDL>
  <Synthesis>
    <Hardware>
      <Component id="reactor" type="reactor" />
      <Component id="separator" type="separator" />
      <Component id="rotavap" type="rotavap" />
      <Component id="cartridge_magnesium_sulfate" type="cartridge" chemical="magnesium sulfate" />
      <Component id="flask_product" type="flask" />
      <Component id="flask_waste" type="flask" />
    </Hardware>
    <Reagents>
      <Reagent name="2-methyl-1,3-cyclohexanedione" role="reagent" solid="True" />
      <Reagent name="potassium hydroxide" role="catalyst" solid="True" />
      <Reagent name="methanol" role="solvent" />
      <Reagent name="argon" role="inert gas" />
      <Reagent name="ethyl vinyl ketone" role="reagent" />
      <Reagent name="saturated aqueous ammonium chloride" role="reagent" />
      <Reagent name="1 M hydrochloric acid" role="acid" />
      <Reagent name="ethyl acetate" role="solvent" />
      <Reagent name="brine" role="reagent" />
      <Reagent name="magnesium sulfate" role="drying agent" />
    </Reagents>
    <Procedure>
      <Add vessel="reactor" reagent="methanol" volume="30 mL" stir="True" />
      <Add vessel="reactor" reagent="potassium hydroxide" amount="89 mg" stir="True" />
      <Add vessel="reactor" reagent="2-methyl-1,3-cyclohexanedione" amount="5.0 g" stir="True" />
      <Purge vessel="reactor" gas="argon" time="5 min" />
      <HeatChillToTemp vessel="reactor" temp="64.7 °C" stir="True" />
      <Add vessel="reactor" reagent="ethyl vinyl ketone" volume="5.54 mL" time="5 min" stir="True"
        stir_speed="300rpm" dropwise="True" />
      <HeatChill vessel="reactor" temp="64.7 °C" time="12 h" stir="True" stir_speed="300 rpm" />
      <HeatChillToTemp vessel="reactor" temp="25 °C" stir="True" />
      <Add vessel="reactor" reagent="saturated aqueous ammonium chloride" volume="30 mL" stir="True" /
>
      <Add vessel="reactor" reagent="1 M hydrochloric acid" volume="30 mL" stir="True" />
      <Transfer from_vessel="reactor" to_vessel="separator" volume="all" />
      <Separate purpose="extract" from_vessel="separator" separation_vessel="separator"
        to_vessel="flask_product"
        product_phase="top" waste_phase_to_vessel="flask_waste" solvent="ethyl acetate"
        solvent_volume="30 mL" />
      <Transfer from_vessel="flask_waste" to_vessel="separator" volume="all" />
      <Separate purpose="extract" from_vessel="separator" separation_vessel="separator"
        to_vessel="flask_product"
        product_phase="top" waste_phase_to_vessel="flask_waste" solvent="ethyl acetate"
        solvent_volume="30 mL" />
      <Transfer from_vessel="flask_waste" to_vessel="separator" volume="all" />
      <Separate purpose="extract" from_vessel="separator" separation_vessel="separator"
        to_vessel="flask_product"
        product_phase="top" waste_phase_to_vessel="flask_waste" solvent="ethyl acetate"
        solvent_volume="30 mL" />
      <Transfer from_vessel="flask_product" to_vessel="separator" volume="all" />
      <Separate purpose="wash" from_vessel="separator" separation_vessel="separator"
        to_vessel="flask_product"
        waste_phase_to_vessel="flask_waste" solvent="brine" solvent_volume="30 mL"
        product_phase="top" />
      <FilterThrough from_vessel="flask_product" to_vessel="rotavap" through="magnesium sulfate" />
      <Evaporate vessel="rotavap" mode="auto" />
    </Procedure>
  </Synthesis>
</XDL>
```

Figure S41: Automatically translated XDL procedure of 2-methyl-2-(3-oxopentyl)-1,3-cyclohexanedione.

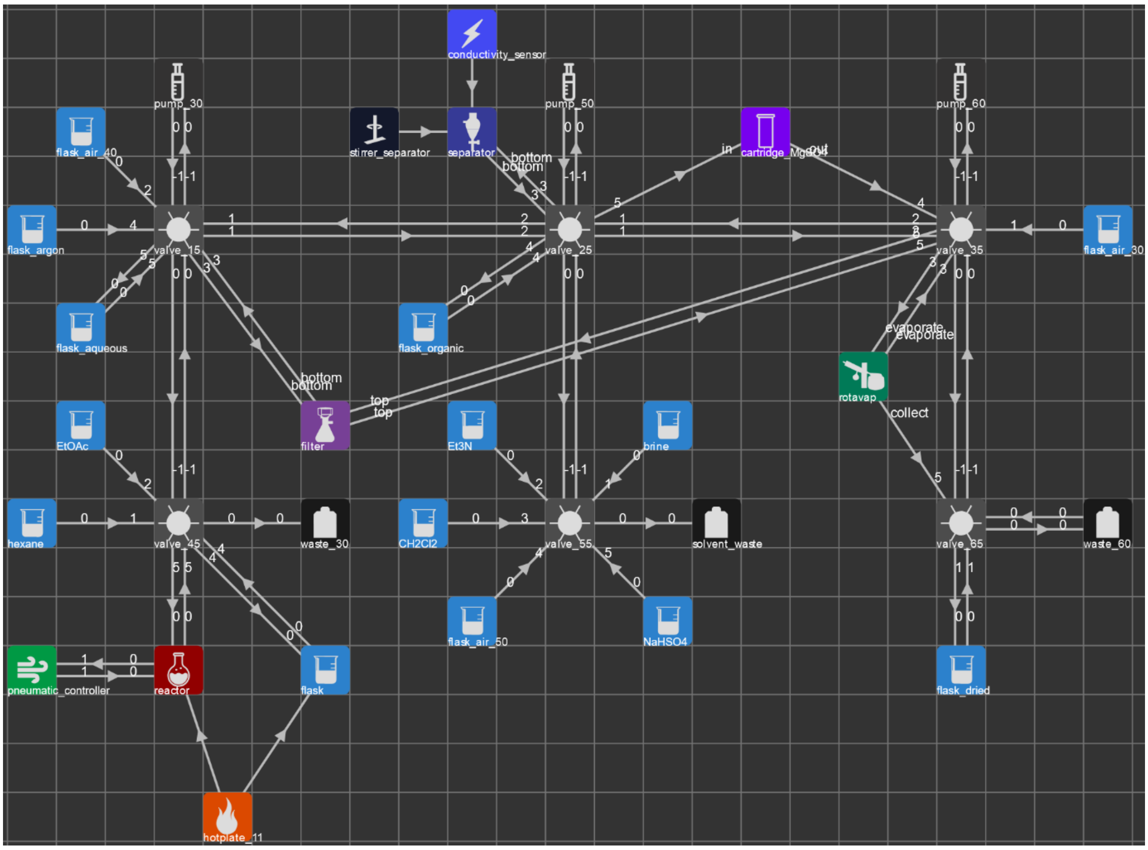

Figure S42: Chemputer graph for procedure of 2-methyl-2-(3-oxopentyl)-1,3-cyclohexanedione.

The product was found in a rotatory evaporator as light yellow oil, 6.24 g, 75% yield.

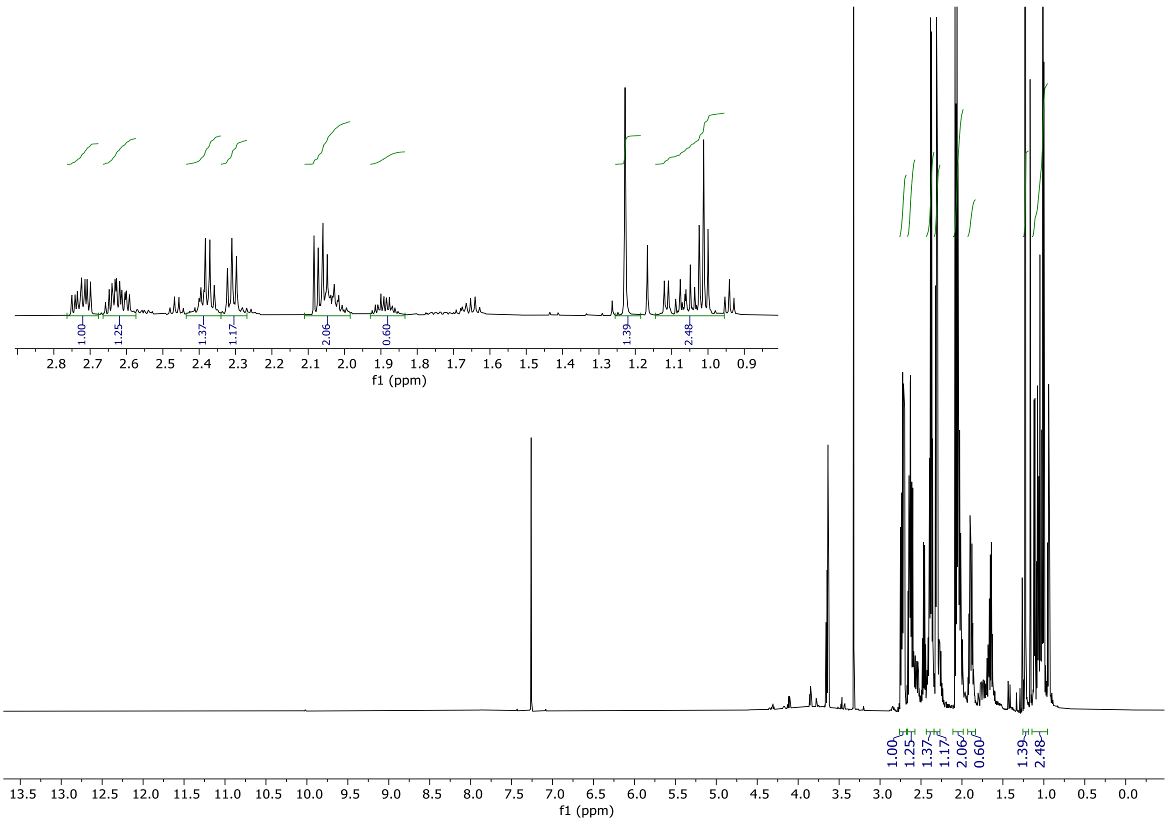

Figure S43: 1H NMR 600 MHz of 2-methyl-2-(3-oxopentyl)-1,3-cyclohexanedione.

5.4 Synthesis of Methyl 4,6-O-benzylidene-α-D-glucopyranoside

Original Procedure:

A mixture of D-glucose (5 g, 27.8 mmol) in dry methanol (16 mL) and deep eutectic solvent (20.0 g) prepared from choline chloride (8.0 g) and malonic acid (12.0 g) was boiled under reflux for 45 min and cooled to room temperature. Benzaldehyde (3.53 g, 3.40 mL, 33.3 mmol) was added and the resulting mixture was heated to 60 °C and stirred for 30 min. After completion of the reaction as indicated by TLC analysis, it was cooled to room temperature and extracted with ethyl acetate (30 × 20 mL). The combined ethyl acetate layer was evaporated under vacuum to afford methyl 4,6-O-benzylidene-α-D-glucopyranoside (2a, 6.2 g, 79.1% yield). The raffinate was treated with water (5.0 mL) and the resulting mass was dehydrated under vacuum on a rotary evaporator at 70 °C for 1 h to afford deep eutectic solvent that could be reused.

Sanitized Procedure:

A mixture of D-glucose (5 g, 27.8 mmol) in dry methanol (16 mL) and deep eutectic solvent (20.0 g) prepared from choline chloride (8.0 g) and malonic acid (12.0 g) was boiled under reflux at 64.7 °C while stirring at 300 rpm for 45 min and then cooled to room temperature (25C). Benzaldehyde (3.53 g, 3.40 mL, 33.3 mmol) was added, and the resulting mixture was heated to 60 °C and stirred at 300 rpm for 30 min. After completion of the reaction as indicated by TLC analysis, it was cooled to room temperature (25 °C) and extracted with ethyl acetate (30 \u00d7 20 mL) using an automatic robotic system. The combined ethyl acetate layer was evaporated under vacuum at 0 mbar to afford methyl 4,6-O-benzylidene-\u03b1-D-glucopyranoside (2a, 6.2 g, 79.1% yield).

```xml
<XDL>
  <Synthesis>
    <Hardware>
      <Component id="reactor1" type="reactor" />
      <Component id="reactor2" type="reactor" />
      <Component id="separator" type="separator" />
      <Component id="rotavap" type="rotavap" />
    </Hardware>
    <Reagents>
      <Reagent name="D-glucose" role="reagent" solid="True" />
      <Reagent name="methanol" role="solvent" />
      <Reagent name="choline chloride" role="reagent" solid="True" />
      <Reagent name="malonic acid" role="reagent" solid="True" />
      <Reagent name="benzaldehyde" role="reagent" />
      <Reagent name="ethyl acetate" role="solvent" />
    </Reagents>
    <Procedure>
      <Add vessel="reactor1" reagent="choline chloride" amount="8 g" stir="True" />
      <Add vessel="reactor1" reagent="malonic acid" amount="12 g" stir="True" />
      <Add vessel="reactor1" reagent="methanol" volume="16 mL" stir="True" />
      <Stir vessel="reactor1" time="20 min" stir_speed="300" />
      <Add vessel="reactor2" reagent="D-glucose" amount="5 g" stir="True" stir_speed="300" />
      <Transfer from_vessel="reactor1" to_vessel="reactor2" volume="all" />
      <Stir vessel="reactor2" time="20 min" stir_speed="300" />
      <HeatChill vessel="reactor2" temp="64.7" time="45 min" stir="True" stir_speed="300" />
      <HeatChillToTemp vessel="reactor2" temp="25" stir="True" />
      <Add vessel="reactor2" reagent="benzaldehyde" volume="3.40 mL" stir="True" />
      <HeatChill vessel="reactor2" temp="60" time="30 min" stir="True" stir_speed="300" />
      <HeatChillToTemp vessel="reactor2" temp="25" stir="True" />
      <Repeat repeats="3">
        <Add vessel="reactor2" reagent="ethyl acetate" volume="20 mL" stir="True" />
        <Transfer from_vessel="reactor2" to_vessel="separator" volume="all" />
        <Separate
          purpose="extract" from_vessel="separator"
          separation_vessel="separator" to_vessel="rotavap"
          product_phase="top" waste_phase_to_vessel="separator" />
      </Repeat>
      <Evaporate vessel="rotavap" mode="auto"/>
    </Procedure>
  </Synthesis>
</XDL>
```

Figure S44: Automatically translated XDL procedure of methyl 4,6-O-benzylidene-α-D-glucopyranoside.

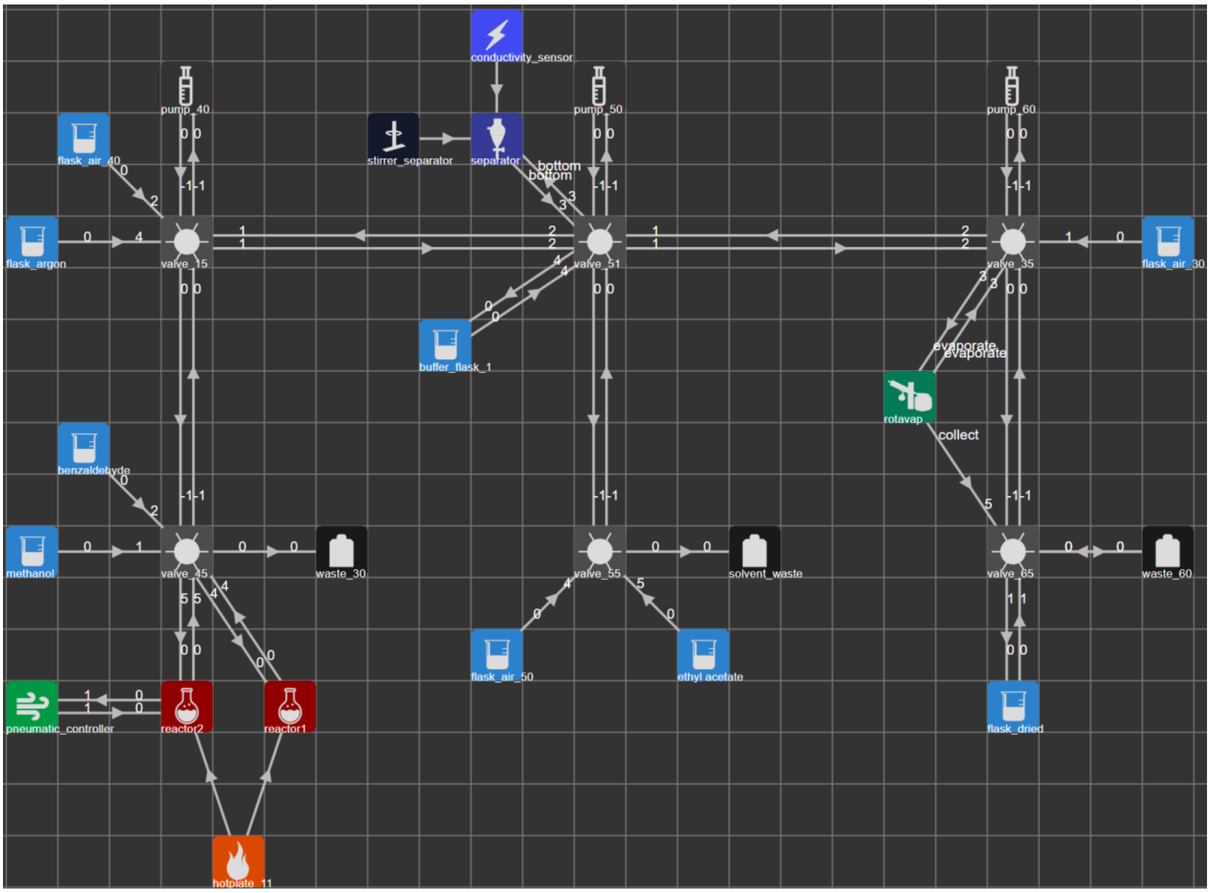

Figure S45: Chemputer graph for procedure of methyl 4,6-O-benzylidene-α-D-glucopyranoside.

The procedure was executed according to the specification twice, however, no conversion was observed. Only the unreacted benzaldehyde was detected in the organic phase. The procedure was repeated 3 times, including pre-drying the eutectic mixture of choline chloride and malonic acid under high vacuum for 4 hours. Even when dried glassware and extra dry methanol were used, no conversion was achieved. The authors validated the translation to XDL as correct. Authors thus concluded that the execution probably requires some specific handling that is not fully captured in the original procedure.

5.5 Synthesis of (2E)-3-[3,4-bis(acetyloxy)phenyl]-2-propenoic acid

Original Procedure:

2.5 Syntheses of coumarate and caffeate derivatives

A solution of caffeic acid (180 mg, 1.0 mmol) in dry pyridine (0.3 mL) was added of acetic anhydride (0.47 mL, 5 mmol) and then stirred at r.t. over night.

The reaction mixture was gently heated under vacuum to evaporate excess acetic anhydride and pyridine thus obtaining quantitatively (2E)-3-[3,4-bis(acetyloxy)phenyl]-2-propenoic acid (264 mg, 1.0 mmol).

Sanitized Procedure:

A solution of caffeic acid (180 mg) in dry pyridine (0.3 mL) was prepared by adding caffeic acid to a vessel, followed by the addition of pyridine while stirring. Acetic anhydride (0.47 mL) was then added to the solution. The reaction mixture was stirred at 25°C for 12 hours. After the reaction period, the mixture was gently heated under reduced pressure to evaporate excess acetic anhydride and pyridine, yielding (2E)-3-[3,4-bis(acetyloxy)phenyl]-2-propenoic acid (264 mg).

```xml
<XDL>
    <Synthesis>
        <Hardware>
            <Component id="reactor_A1"
                       type="reactor" />
        </Hardware>
        <Reagents>
            <Reagent name="caffeic acid"
                     role="substrate"
                     solid="True" />
            <Reagent name="pyridine"
                     role="solvent" />
            <Reagent name="acetic anhydride"
                     role="reagent" />
        </Reagents>
        <Procedure>
            <Add vessel="reactor_A1"
                 reagent="caffeic acid"
                 amount="180 mg"
                 stir="True" />
            <Add vessel="reactor_A1"
                 reagent="pyridine"
                 volume="0.3 mL"
                 stir="True" />
            <Add vessel="reactor_A1"
                 reagent="acetic anhydride"
                 volume="0.47 mL"
                 stir="True" />
            <HeatChill vessel="reactor_A1"
                       temp="25"
                       time="12 h"
                       stir="True"
                       stir_speed="300" />
            <StopHeatChill vessel="reactor_A1" />
        </Procedure>
    </Synthesis>
</XDL>
```

Figure S46: Generated XDL for the synthesis of (2E)-3-[3,4-bis(acetyloxy)phenyl]-2-propenoic acid.

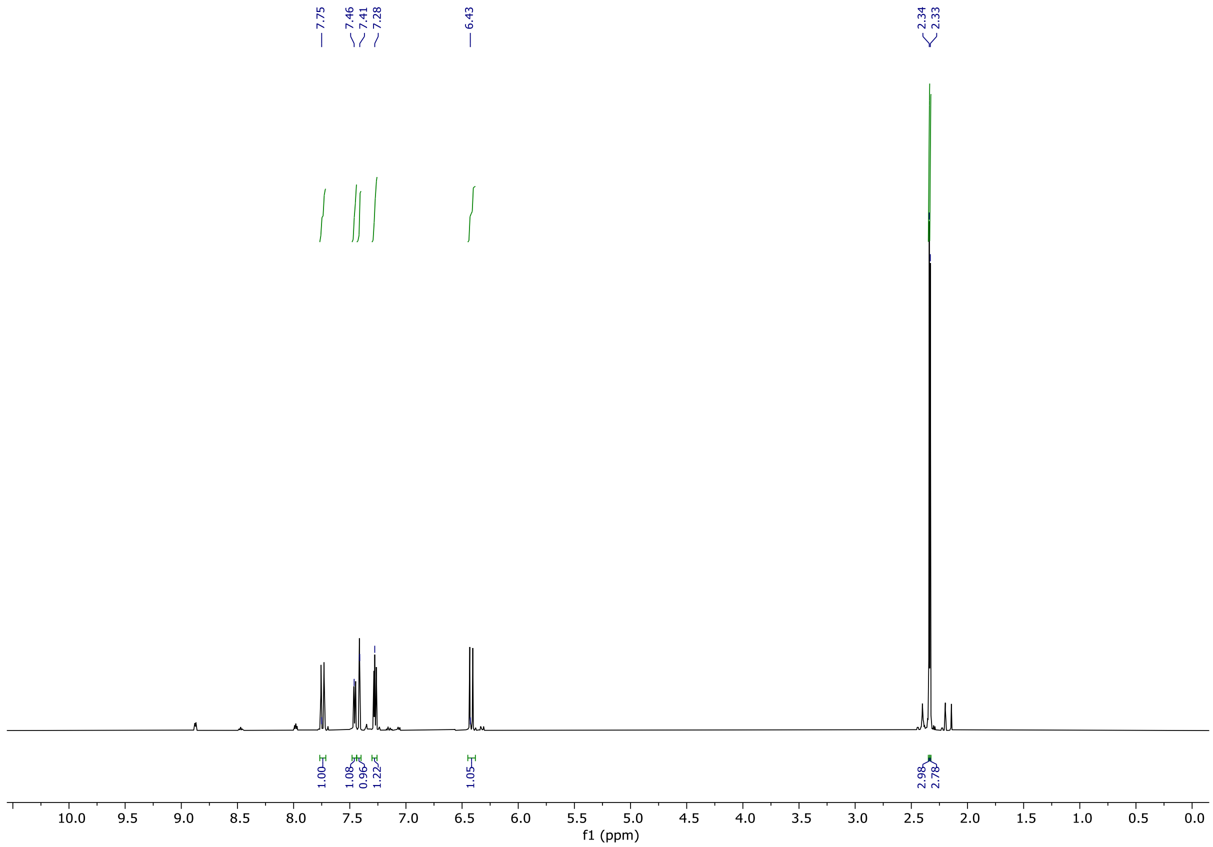

Figure S47: 1H NMR 600 MHz of (2E)-3-[3,4-bis(acetyloxy)phenyl]-2-propenoic acid

5.6 Synthesis of 4-(4-nitrophenyl)morpholine

Original Procedure:

Step 1: 4-(4-Nitro-phenyl)-morpholine (161-a) 1-Fluoro-4-nitrobenzene (3.00 mL, 28.3 mmol) was combined in a flask with anhydrous 2-propanol (28 mL), triethylamine (4.34 mL, 311 mmol), and morpholine (2.47 mL, 28.3 mmol). The mixture was refluxed for 3.5 h before adding additional morpholine (2.47 mL, 28.3 mmol) and refluxing for 20 h. The mixture was then cooled and concentrated under reduced pressure. Purification by silica gel flash chromatography provided 161-a (6.09 g, 98%) as a 95% pure solid.

Sanitized Procedure:

1. In a suitable reaction flask, combine 3.00 mL of 1-Fluoro-4-nitrobenzene (28.3 mmol), 28 mL of anhydrous 2-propanol, 4.34 mL of triethylamine (311 mmol), and 2.47 mL of morpholine (28.3 mmol). 2. Heat the mixture to reflux at 82.5°C while stirring for 3.5 hours. 3. Add an additional 2.47 mL of morpholine (28.3 mmol) to the reaction mixture. 4. Continue refluxing the mixture at 82.5°C for an additional 20 hours. 5. After the reflux period, cool the reaction mixture to room temperature (25°C). 6. Concentrate the mixture under reduced pressure (0 mbar) to remove the solvent. 7. Purify the residue by silica gel flash chromatography to obtain the product, 4-(4-Nitro-phenyl)-morpholine (161-a).

```
<XDL>
    <Synthesis>
        <Hardware>
            <Component id="vessel1"
                       type="reactor" />
            <Component id="vessel2"
                       type="flask"
                       chemical="residue" />
        </Hardware>
        <Reagents>
            <Reagent name="1-Fluoro-4-nitrobenzene"
                     role="reagent" />
            <Reagent name="2-propanol"
                     role="solvent" />
            <Reagent name="triethylamine"
                     role="base" />
            <Reagent name="morpholine"
                     role="reagent" />
        </Reagents>
        <Procedure>
            <Add vessel="vessel1"
                 reagent="1-Fluoro-4-nitrobenzene"
                 volume="3.00 mL"
                 stir="True"
                 stir_speed="300" />
            <Add vessel="vessel1"
                 reagent="2-propanol"
                 volume="28 mL"
                 stir="True"
                 stir_speed="300" />
            <Add vessel="vessel1"
                 reagent="triethylamine"
                 volume="4.34 mL"
                 stir="True"
                 stir_speed="300" />
            <Add vessel="vessel1"
                 reagent="morpholine"
                 volume="2.47 mL"
                 stir="True"
                 stir_speed="300" />
            <HeatChill vessel="vessel1"
                       temp="82.5"
                       time="3.5 h"
                       stir="True"
                       stir_speed="300" />
            <Add vessel="vessel1"
                 reagent="morpholine"
                 volume="2.47 mL"
                 stir="True"
                 stir_speed="300" />
            <HeatChill vessel="vessel1"
                       temp="82.5"
                       time="20 h"
                       stir="True"
                       stir_speed="300" />
            <HeatChillToTemp vessel="vessel1"
                             temp="25"
                             active="True" />
            <Transfer from_vessel="vessel1"
                      to_vessel="vessel2"
                      volume="all" />
        </Procedure>
    </Synthesis>
</XDL>
```

Figure S48: Generated XDL for the synthesis of 4-(4-nitrophenyl)morpholine.

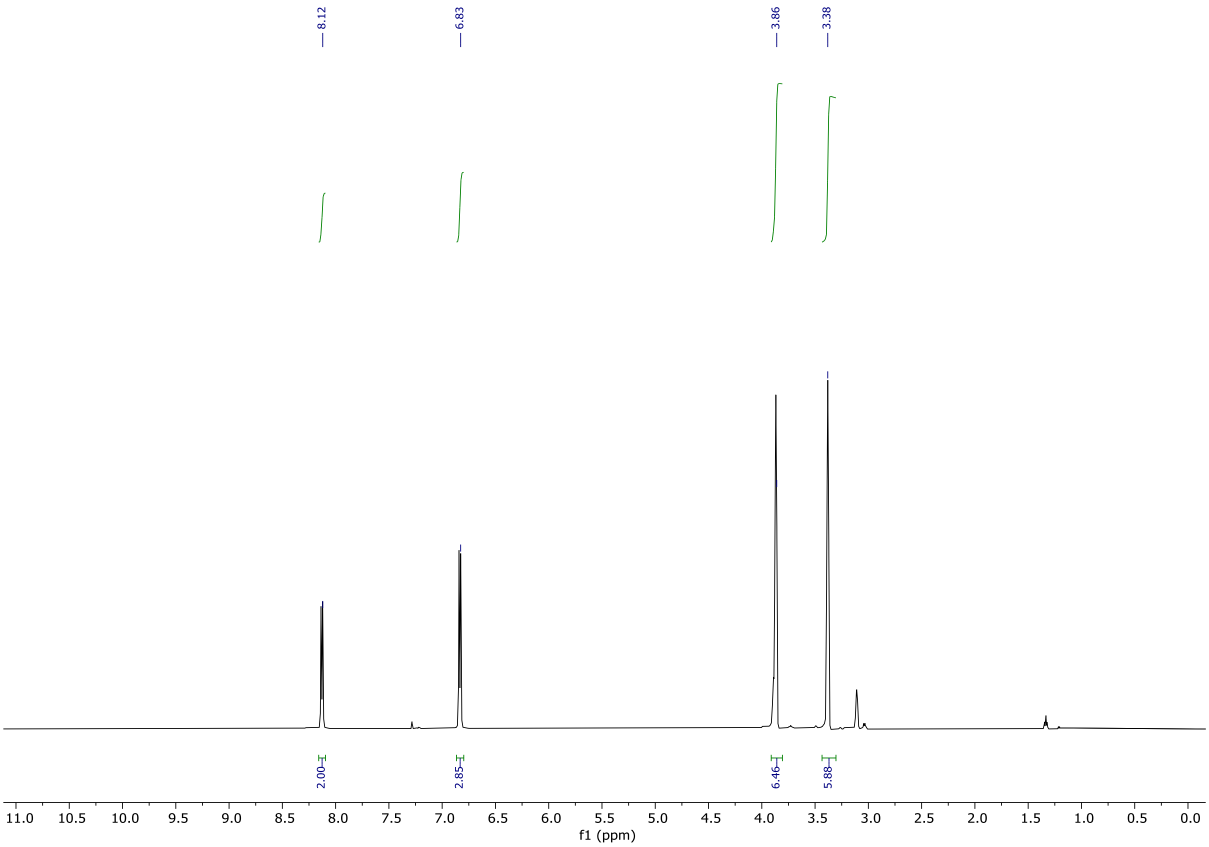

Figure S49: 1H NMR 600 MHz of 4-(4-nitrophenyl)morpholine

References

1. properties of common organic compounds - sortable. http://murov.info/orgcmpds.htm.
2. Bran, A. M., Cox, S., White, A. D. & Schwaller, P. ChemCrow: Augmenting large-language models with chemistry tools. Preprint at http://arxiv.org/abs/2304.05376 (2023).
3. Prince, M. H. *et al.* Opportunities for Retrieval and Tool Augmented Large Language Models in Scientific Facilities.
4. Skreta, M. *et al.* Errors are Useful Prompts: Instruction Guided Task Programming with Verifier-Assisted Iterative Prompting. Preprint at http://arxiv.org/abs/2303.14100 (2023).
5. XDL 2.0 Standard — xdl 2.0.1.dev12+g8fdf586b documentation. https://croningroup.gitlab.io/chemputer/xdl/standard/index.html.
6. Yao, S. *et al.* ReAct: Synergizing Reasoning and Acting in Language Models. Preprint at https://doi.org/10.48550/arXiv.2210.03629 (2023).
7. Rohrbach, S. *et al.* Digitization and validation of a chemical synthesis literature database in the ChemPU. *Science* **377**, 172–180 (2022).
8. Zheng, L. *et al.* Judging LLM-as-a-Judge with MT-Bench and Chatbot Arena. Preprint at https://doi.org/10.48550/arXiv.2306.05685 (2023).
9. M Bran, A., Jončev, Z. & Schwaller, P. Knowledge Graph Extraction from Total Synthesis Documents. in *Proceedings of the 1st Workshop on Language + Molecules (L+M 2024)* 74–84 (Association for Computational Linguistics, Bangkok, Thailand, 2024). doi:10.18653/v1/2024.langmol-1.9.
10. Kearnes, S. M. *et al.* The Open Reaction Database. *J. Am. Chem. Soc.* **143**, 18820–18826 (2021).
11. Fan, V. *et al.* OpenChemIE: An Information Extraction Toolkit For Chemistry Literature. Preprint at https://doi.org/10.48550/arXiv.2404.01462 (2024).
12. Patiny, L. & Godin, G. Automatic extraction of FAIR data from publications using LLM. Preprint at https://doi.org/10.26434/chemrxiv-2023-05v1b (2023).

13. Ai, Q., Meng, F., Shi, J., Pelkie, B. & Coley, C. W. Extracting structured data from organic synthesis procedures using a fine-tuned large language model. *Digit. Discov.* **3**, 1822–1831 (2024).
14. Ai, Q., Meng, F., Shi, J., Pelkie, B. & Coley, C. W. Extracting structured data from organic synthesis procedures using a fine-tuned large language model. *Digit. Discov.* **3**, 1822–1831 (2024).
15. Mavračić, J., Court, C. J., Isazawa, T., Elliott, S. R. & Cole, J. M. ChemDataExtractor 2.0: Autopopulated Ontologies for Materials Science. *J. Chem. Inf. Model.* **61**, 4280–4289 (2021).
16. Swain, M. C. & Cole, J. M. ChemDataExtractor: A Toolkit for Automated Extraction of Chemical Information from the Scientific Literature. *J. Chem. Inf. Model.* **56**, 1894–1904 (2016).
17. Mehr, S. H. M., Craven, M., Leonov, A. I., Keenan, G. & Cronin, L. A universal system for digitization and automatic execution of the chemical synthesis literature. *Science* **370**, 101–108 (2020).
18. Brown, T. B. *et al.* Language Models are Few-Shot Learners. Preprint at http://arxiv.org/abs/2005.14165 (2020).
19. DeepSeek-AI *et al.* DeepSeek-V3 Technical Report. Preprint at https://doi.org/10.48550/arXiv.2412.19437 (2024).
20. Grattafiori, A. *et al.* The Llama 3 Herd of Models. Preprint at https://doi.org/10.48550/arXiv.2407.21783 (2024).
21. ollama/ollama: Get up and running with Llama 3.3, DeepSeek-R1, Phi-4, Gemma 2, and other large language models. *GitHub* https://github.com/ollama/ollama.
22. Elsevier Information Systems GmbH. Reaxys. (2024).
23. Foundations of Statistical Natural Language Processing. https://nlp.stanford.edu/fsnlp/.
24. Steiner, S. *et al.* Organic synthesis in a modular robotic system driven by a chemical programming language. *Science* **363**, eaav2211 (2019).
25. Rauschen, R., Guy, M., Hein, J. E. & Cronin, L. Universal chemical programming language for robotic synthesis repeatability. *Nat. Synth.* **3**, 488–496 (2024).
